# Dromedary camel nanobodies broadly neutralize SARS-CoV-2 variants

**DOI:** 10.1073/pnas.2201433119

**Published:** 2022-04-27

**Authors:** Jessica Hong, Hyung Joon Kwon, Raul Cachau, Catherine Z. Chen, Kevin John Butay, Zhijian Duan, Dan Li, Hua Ren, Tianyuzhou Liang, Jianghai Zhu, Venkata P. Dandey, Negin P. Martin, Dominic Esposito, Uriel Ortega-Rodriguez, Miao Xu, Mario J. Borgnia, Hang Xie, Mitchell Ho

**Affiliations:** ^a^Laboratory of Molecular Biology, Center for Cancer Research, National Cancer Institute, NIH, Bethesda, MD 20891;; ^b^Division of Viral Products, Office of Vaccines Research and Review, Center for Biologics Evaluation and Research, United States Food and Drug Administration, Silver Spring, MD 20993;; ^c^Data Science and Information Technology Program, Leidos Biomedical Research, Inc., Frederick, MD 21702;; ^d^National Center for Advancing Translational Sciences, NIH, Rockville, MD 20850;; ^e^Molecular Microscopy Group, National Institute of Environmental Health Sciences, Research Triangle Park, NC 27709;; ^f^Antibody Engineering Program, Center for Cancer Research, National Cancer Institute, NIH, Bethesda, MD 20891;; ^g^Viral Vector Core, National Institute of Environmental Health Sciences, Research Triangle Park, NC 27709;; ^h^Protein Expression Laboratory, NCI RAS Initiative, Frederick National Laboratory for Cancer Research, Frederick, MD 21702

**Keywords:** SARS-CoV-2, single-domain antibody, dromedary camel nanobody VHH, neutralizing antibody, cryo-EM

## Abstract

The severe acute respiratory syndrome coronavirus 2 (SARS-CoV-2) spike is a trimer of S1/S2 heterodimers with three receptor-binding domains (RBDs) at the S1 subunit for human angiotensin-converting enzyme 2 (hACE2). Due to their small size, nanobodies can recognize protein cavities that are not accessible to conventional antibodies. To isolate high-affinity nanobodies, large libraries with great diversity are highly desirable. Dromedary camels (*Camelus dromedarius*) are natural reservoirs of coronaviruses like Middle East respiratory syndrome CoV (MERS-CoV) that are transmitted to humans. Here, we built large dromedary camel V_H_H phage libraries to isolate nanobodies that broadly neutralize SARS-CoV-2 variants. We isolated two V_H_H nanobodies, NCI-CoV-7A3 (7A3) and NCI-CoV-8A2 (8A2), which have a high affinity for the RBD via targeting nonoverlapping epitopes and show broad neutralization activity against SARS-CoV-2 and its emerging variants of concern. Cryoelectron microscopy (cryo-EM) complex structures revealed that 8A2 binds the RBD in its up mode with a long CDR3 loop directly involved in the ACE2 binding residues and that 7A3 targets a deeply buried region that uniquely extends from the S1 subunit to the apex of the S2 subunit regardless of the conformational state of the RBD. At a dose of ≥5 mg/kg, 7A3 efficiently protected transgenic mice expressing hACE2 from the lethal challenge of variants B.1.351 or B.1.617.2, suggesting its therapeutic use against COVID-19 variants. The dromedary camel V_H_H phage libraries could be helpful as a unique platform ready for quickly isolating potent nanobodies against future emerging viruses.

## Significance

Due to their small size, nanobodies can recognize protein cavities that are not accessible to conventional antibodies. In this report, we built dromedary camel (*Camelus dromedarius*) V_H_H phage libraries for the isolation of high-affinity nanobodies that broadly neutralize SARS-CoV-2 variants. Cryo-EM complex structures reveal that one dromedary camel V_H_H nanobody (8A2) binds the S1 subunit of the viral spike protein, and the other (7A3) targets a deeply buried region that uniquely extends to the S2 subunit beyond the S1 subunit. These nanobodies can protect mice from the lethal challenge of variants B.1.351 or B.1.617.2, suggesting the therapeutic potential of these nanobodies against COVID-19. The dromedary camel V_H_H libraries could be helpful to isolate neutralizing nanobodies against future emerging viruses quickly.

Severe acute respiratory syndrome coronavirus 2 (SARS-CoV-2) is the etiologic agent of COVID-19 (1, 2) that enters human cells by binding its envelope anchored type I fusion protein (spike) to angiotensin-converting enzyme 2 (ACE2) (3, 4). The SARS-CoV-2 spike is a trimer of S1/S2 heterodimers with three ACE2 receptor-binding domains (RBDs) attached to the distal end of the spike via a hinge region that allows conformational flexibility (4). In the all-down conformation, the RBDs are packed with their long axes contained in a plane perpendicular to the axis of symmetry of the trimer. Transition to the roughly perpendicular up conformation exposes the receptor-binding motif (RBM), located at the distal end of the RBD, which is sterically occluded in the down state. Numerous neutralizing antibodies targeting the spike, particularly its RBD, have been developed to treat COVID-19 using common strategies such as single B cell cloning, animal immunization, and phage display (5–9). Most vaccines, including those that are messenger RNA based, are designed to induce immunity against the spike or RBD (10–12). However, emerging SARS-CoV-2 variants such as D614G, B.1.1.7 (Alpha, United Kingdom), B.1.351 (Beta, South Africa), and P.1 (Gamma, Brazil) have exhibited increased resistance to neutralization by monoclonal antibodies or postvaccination sera elicited by the COVID-19 vaccines (13, 14). Monoclonal antibodies with Emergency Use Authorization for COVID-19 treatment partially (Casirivimab) or completely (Bamlanivimab) failed to inhibit the B.1.351 and P.1 variants. Similarly, these variants were less effectively inhibited by convalescent plasma and sera from individuals vaccinated with a COVID-19 vaccine (BNT162b2) (13). The B.1.617.2 (Delta, India) variant became the prevailing strain in many countries (15). Highly effective and broadly neutralizing antibody therapy is urgently demanded for COVID-19 patients.

Due to their small size and unique conformations, camelid V_H_H single-domain antibodies (also known as nanobodies) can recognize protein cavities that are not accessible to conventional antibodies (16). To isolate high-affinity nanobodies without a need for further affinity maturation, it is highly desirable to construct large nanobody libraries with great diversity. Dromedary camels have been found as potential natural reservoirs of Middle East respiratory syndrome CoV (MERS-CoV) (17). We speculated that dromedary camels would be an ideal source of neutralizing nanobodies against coronaviruses. In the present study, we built large camel V_H_H single-domain antibody phage libraries with a diversity of over 10^11^ from six dromedary camels (*Camelus dromedarius*), three males and three females, with ages ranging from 3 mo to 20 y. We used both the SARS-CoV-2 RBD and the stabilized spike ectodomain trimer protein as baits to conduct phage panning for nanobody screening. Among all the binders, we found NCI-CoV-7A3 (7A3), NCI-CoV-1B5 (1B5), NCI-CoV-8A2 (8A2), and NCI-CoV-2F7 (2F7) to be potent ACE2 blockers. In addition, these dromedary camel nanobodies displayed potent neutralization activity against the B.1.351 and B.1.1.7 variants and the original strain (Wuhan-Hu-1). The cryoelectron microscopy (cryo-EM) structure of the spike trimer protein complex with these V_H_H nanobodies revealed two distinct nonoverlapping epitopes for neutralizing SARS-CoV-2. In particular, 7A3 recognizes a unique and deeply buried region that extends to the apex of the S2 subunit of the spike. Combined treatment with 7A3 and 8A2 shows more potent protection against various variants in culture and mice infected with the B.1.351 variant. Interestingly, 7A3 alone retains its neutralization activity against the lethal challenge of the B.1.617.2 variant in mice.

## Results

### Isolation of High-Affinity Dromedary Camel Nanobodies against SARS-CoV-2.

**Fig. 1.**
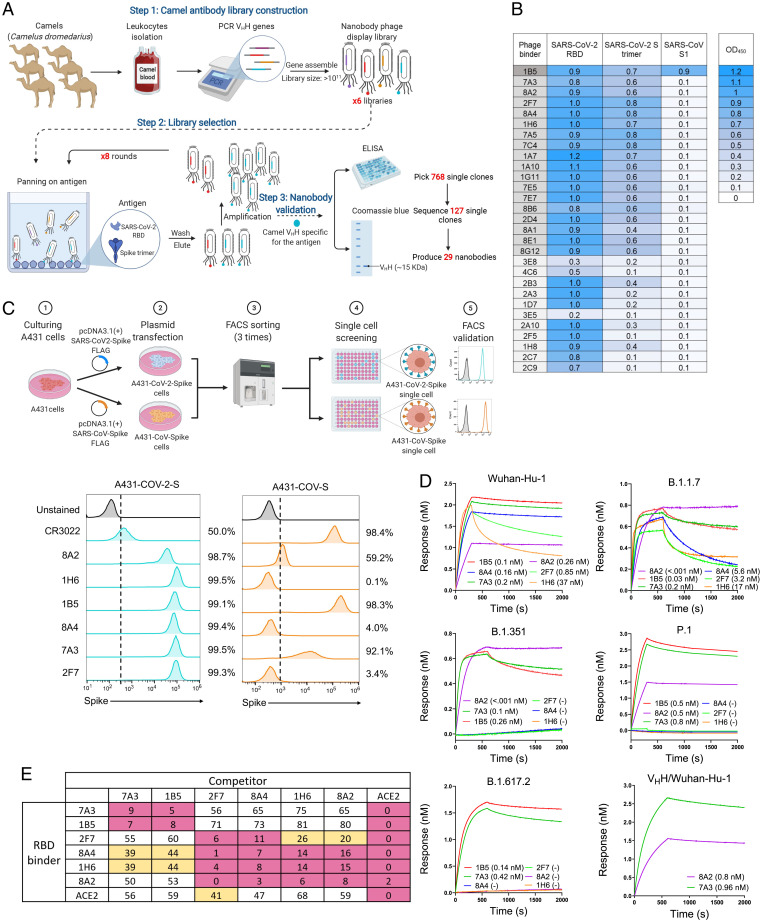
Isolation of high-affinity camel nanobodies against SARS-CoV-2. (*A*) Isolation of camel V_H_Hs that bind the RBD by phage display. (*B*) Camel V_H_Hs against the S protein of SARS-CoV-2 or SARS-CoV. (*C*) Flow cytometry (FACS) was performed to monitor the cross-reaction of nanobodies to the spike of both SARS-CoV-2 and SARS-CoV on human cells. The cartoon outlines the experimental workflow for overexpression of SARS-CoV-2-spike or SARS-CoV-spike in the A431 human cell line. Both cell lines were stained with V_H_H nanobodies or CR3022 as a positive control. (*D*) Binding (K_D_) of V_H_H-hFc or V_H_Hs against the RBD of SARS-CoV-2 and its variants. (*E*) Cross-competition assay of each nanobody and ACE2 on Octet.

To identify nanobodies against SARS-CoV-2, we screened the RBD or the stabilized S protein (18) with prolines substituted at residues 986 and 987 using our V_H_H single domain phage display libraries constructed from six camels (three males and three females) with ages ranging from 3 mo to 20 y (Fig. 1*A*). In total, we isolated 768 V_H_H phage clones; among them, 127 V_H_H clones had a high binding signal for the RBD in enzyme-linked immunosorbent assay (ELISA). Out of the 127 clones, there were 29 unique V_H_H sequences (Fig. 1*B*). We selected the top six V_H_H single domains that bind both the RBD and the S protein of SARS-CoV-2. The 1B5 also binds the S1 subunit of SARS-CoV, previously detected in 2003 (5, 18). The sequence alignment of the top six sequences showed that 7A3 and 8A4 contain only two canonical cysteines, one N-terminal to the CDR1 and the other before CDR3 (*SI Appendix*, Fig. S1). The remaining V_H_Hs, 1B5, 8A2, 2F7, and 1H6, have a total of four cysteines with two additional, noncanonical cysteines, one in the CDR1 and the other in the CDR3. The nanobodies with four cysteines are common in camels and sharks (19–21).

To validate the binding and cross-reactivity of the anti-RBD specific V_H_H nanobodies, we engineered two human cell lines, referred to as “A431-CoV-2-S” and “A431-CoV-S,” which overexpressed the S protein of either SARS-CoV-2 or SARS-CoV, respectively, on the cell surface (Fig. 1*C*). The previously characterized CR3022 antibody was used as a control antibody due to its cross-reactivity to both SARS-CoV and SARS-CoV-2 RBD (22). All six anti-RBD nanobodies could bind A431-CoV-2-S cell lines with high affinity. Two V_H_Hs (1B5 and 7A3) showed cross-reactivity to A431-CoV-S, with 8A2 having a modest signal (Fig. 1*C*). These results indicate that the identified camel V_H_H nanobodies can bind the S protein of SARS-CoV-2 on human cell surface.

We carried out an Octet analysis to examine the binding property against wild-type SARS-CoV-2 and emerging variants, B.1.1.7, B.1.351, P.1, and B.1.617.2 (Fig. 1*D* and *SI Appendix*, Fig. S2 and Table S1). All six V_H_H-hFc fusion proteins exhibited subnanomolar binding avidity to Wuhan-Hu-1 and the B.1.1.7 variant, indicating all six camel V_H_Hs can tolerate the D614G and N501Y mutations in B.1.1.7. For the B.1.351 and P.1 variants, only 8A2 (0.001 nM, 0.5 nM), 7A3 (0.1 nM, 0.8 nM), and 1B5 (0.26 nM, 0.5 nM) exhibited strong binding to the spike protein, whereas the rest of the V_H_Hs lost binding for these two variant spikes. The Octet data suggest that the K417N/T and E484K mutations in B.1.351 and P1 do not affect the binding of 8A2, 7A3, and 1B5 to the RBD. Regarding B.1.617.2, only 1B5 (0.14 nM) and 7A3 (0.42 nM) retained binding, whereas 8A2 lost most of its binding capacity to this variant, indicating L452R in B.1.617.2 might affect 8A2 binding to the spike. To measure the binding affinity, we expressed monomeric camel V_H_Hs with a six-histidine tag in *Escherichia coli* and purified them on a nickel column. Using the V_H_H concentrations ranging from 12.5 nM to 100 nM, we measured the binding affinity values (*K*_D_) of the monomeric 7A3 (0.96 nM), 8A2 (0.8 nM), and 2F7 (0.75 nM) V_H_Hs (Fig. 1*D* and *SI Appendix*, Fig. S3). Furthermore, a cross-competition assay showed two distinct epitope groups: 7A3 and 1B5 bind to a similar epitope, whereas the remaining nanobodies (2F7, 8A4, 1H6, and 8A2) bind to a different region, indicating two nonoverlapping epitopes on the RBD (Fig. 1*E*). Epitope mapping using an RBD-derived peptide array indicates that the nanobodies bind discontinuous conformation epitopes (*SI Appendix*, Fig. S4). Additionally, we examined the inhibitory effect of V_H_H single domains against the RBD–human ACE2 interaction (*SI Appendix*, Fig. S5). The 1B5 nanobody (concentration that inhibits response by 50% [IC_50_] 3.2 nM) was the most potent ACE2 inhibitor, followed by 8A2 (IC_50_ 8 nM). Taken together, we isolated the camel nanobodies that bind two distinct epitopes on the RBD with high affinity. One epitope recognized by 7A3 and 1B5 is conserved between SARS-CoV-2 and SARS-CoV; the other is recognized by 8A2 and 2F7 and is unique for SARS-CoV-2.

### Dromedary Camel Nanobodies Neutralize SARS-CoV-2 Variants.

**Fig. 2.**
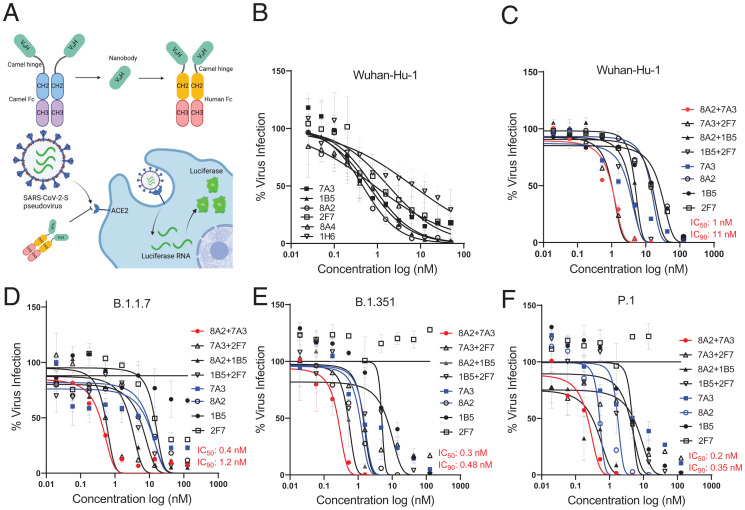
Nanobodies neutralize SARS-CoV-2 and the variants in pseudovirus assay. (*A*) Diagrams illustrating pseudovirus assay and V_H_H-hFc. (*B*) Camel V_H_H-hFc proteins inhibit SARS-CoV-2 pseudovirus infectivity to ACE2 expressing human cells by measuring luciferase expression. (*C*–*F*) Pseudovirus particle neutralization assay testing 2-in-1 combinations and single nanobodies showing that 7A3 + 8A2 combination has the best neutralization activity.

To determine whether the top six nanobodies can neutralize SARS-CoV-2 entry, we tested them in pseudovirus neutralization assays in multiple systems, using V_H_H-hFc fusions (Fig. 2*A*). Among the top six V_H_Hs, 8A2 had the highest inhibitory activity against the Wuhan-Hu-1 pseudovirus, with an IC_50_ of 5 nM (Fig. 2*B*). We also used a microneutralization assay to prescreen the top six V_H_Hs against the original strain and the D614G variant. We found that 8A2 showed the most neutralizing activity against early SARS-CoV-2 with or without D614G (*SI Appendix*, Fig. S6). This result was consistent with the pseudovirus neutralization result in Fig. 2*B*, suggesting that 8A2 was more potent in blocking the entry of early SARS-CoV-2 than any of the other five V_H_Hs. We then tested 2-in-1 and 3-in-1 combinations for all six binders in the pseudovirus infection assay. We found that the combination of 7A3 + 8A2 displayed the highest efficacy (IC_50_ of 1.6 nM or 2.3 nM) compared to the other combinations or the individual nanobodies in both pseudovirus infection and reporter assays (*SI Appendix*, Fig. S7 and Table S2). We also tested the combinations of 3-in-1 V_H_Hs and did not find that they further improved the efficacy. During the process of characterizing these V_H_H nanobodies, several variants of concern (VOCs) emerged, including B.1.1.7 (Alpha), B.1.351 (Beta), and P.1 (Gamma). We tested the top four neutralizing nanobodies (7A3, 8A2, 1B5, and 2F7) for the original Wuhan-Hu-1 on these pseudovirus variants to assess whether our nanobodies could neutralize these VOCs as single agents and combinations (Fig. 2 *C*–*F*). We found that the combination of 7A3 and 8A2 demonstrated the best efficacy in neutralizing Wuhan-Hu-1 and the B.1.1.7, B.1.351, and P.1 variants, with an IC_50_ of 1, 0.4, 0.3, and 0.2 nM, respectively (Fig. 2 *C*–*F* and *SI Appendix*, Table S2). Furthermore, fluorescence reporter-based pseudovirus data validated luciferase-based pseudovirus data about the potency of the combination of 7A3 and 8A2 (*SI Appendix*, Fig. S8). Together, our camel nanobodies exhibit potent neutralization activity against B.1.351, B.1.1.7, and P.1 variants, with the combination of 7A3 and 8A2 showing the highest level of efficacy.

**Fig. 3.**
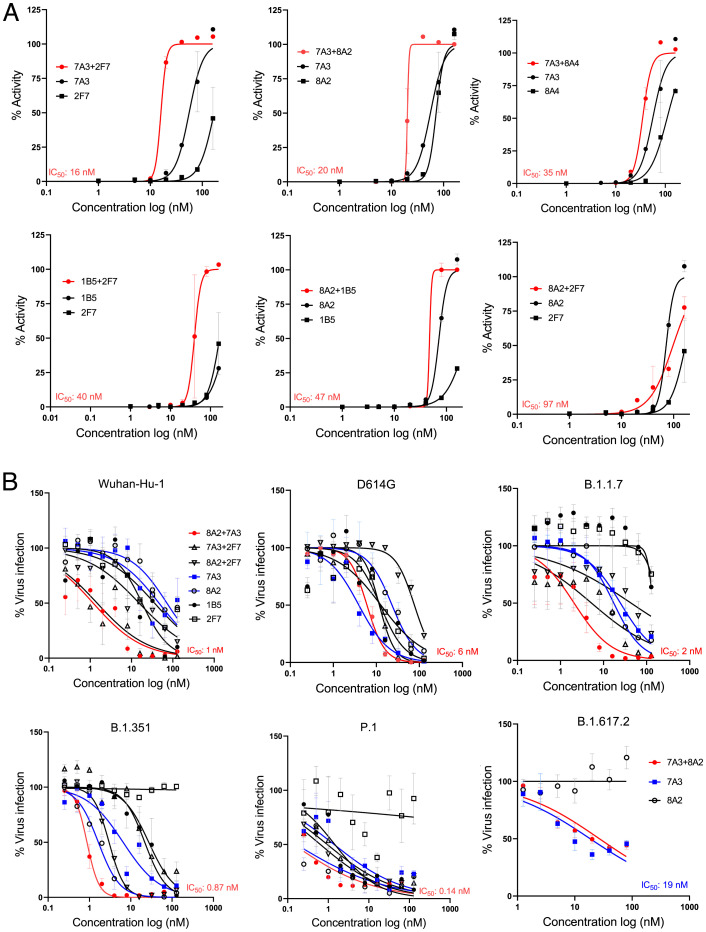
Neutralization of SARS-CoV-2 and its variants in live virus assay. (*A*) Live virus neutralization assay of nanobodies 7A3, 8A2, 2F7, 1B5, and 8A4 along with their 2-in-1 combinations against Wuhan-Hu-1. (*B*) Live variant virus assay using the top three nanobody combinations of 8A2 + 7A3, 8A2 + 2F7, and 7A3 + 2F7 was conducted against Wuhan-Hu-1, D614G, B.1.1.7, B.1.351, P.1, and B.1.617.2.

To determine the most effective single-domain antibody combination against the live virus, we tested different combinations of 7A3, 2F7, 1B5, 8A2, and 8A4 V_H_H-hFc fusion proteins along with their single agents, using a cytopathic effect (CPE) assay. Among them, 7A3 + 2F7 and 7A3 + 8A2 displayed the best activity, with IC_50_ of 16 and 20 nM, respectively (Fig. 3*A* and *SI Appendix*, Table S3). To further test the combinations against variant viruses, we used three 2-in-1 combinations (8A2 + 7A3, 8A2 + 2F7, and 7A3 + 2F7) in a live virus–based microneutralization assay against the early SARS-CoV-2 strain harboring the D614G mutation, and the VOCs including B.1.1.7, B.1.351, P.1, and B.1.617.2. In general, the combination of 8A2 and 7A3 showed potent neutralization activity across all virus variants tested, with IC_50_ values of 6 nM (D614G), 2 nM (B.1.1.7), 0.87 nM (B.1.351), 0.14 nM (P.1), and 27 nM (B.1.617.2) (Fig. 3*B* and *SI Appendix*, Table S3). While 8A2 did not neutralize B.1.617.2, 7A3 alone exhibited better neutralization activity (IC_50_ 19 nM) than the 2-in-1 combination (IC_50_ 27 nM) against this variant (Fig. 3*B* and *SI Appendix*, Table S3). These results indicate that 7A3 has broad neutralization activity against all variants tested, including B.1.617.2.

### Dromedary Camel Nanobodies Protect Mice from Lethal SARS-CoV-2 Infection.

**Fig. 4.**
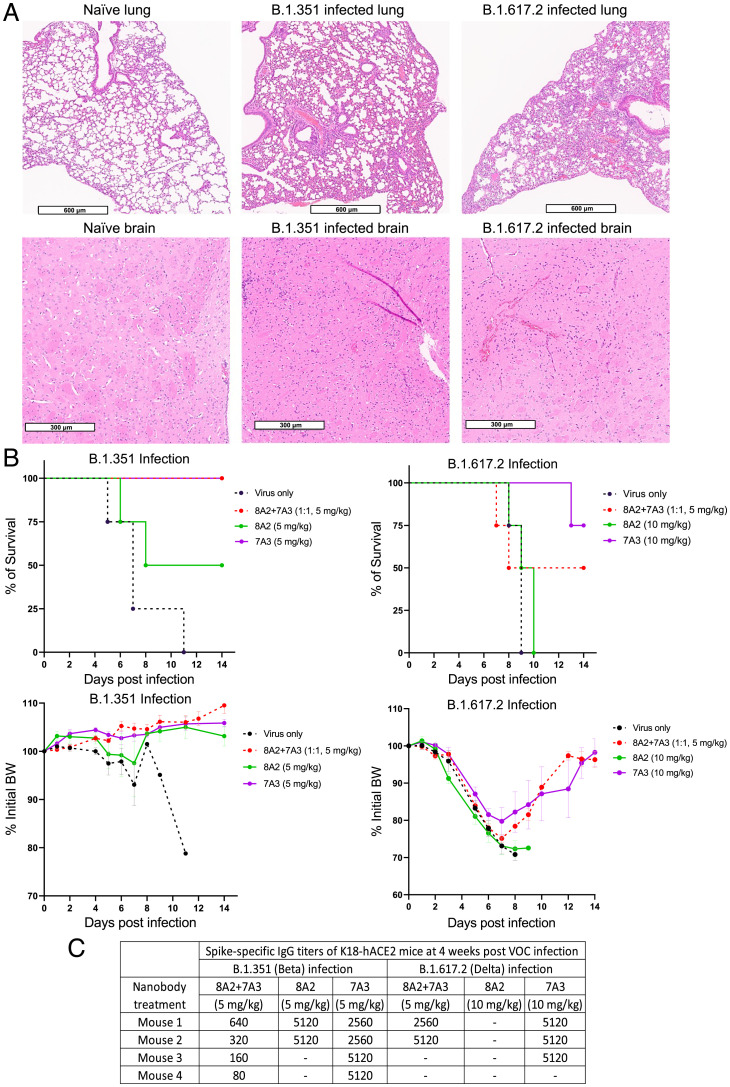
Protection of the K18-hACE2 mice infected with a lethal dose of B.1.351 or B.1.617.2 variant. (*A*) The histology of lung and brain tissues harvested from K18-hACE2 transgenic mice on day 6 or 7 following infection of live SARS-CoV-2 B.1.351 or B.1.617.2 variant virus. (*B*) K18-hACE2 mice (*n* = 4 per group) were injected intraperitoneally with nanobody 7A3, 8A2, or 2-in-1 mixture at indicated doses followed by a lethal infection of B.1.351 or B.1.617.2 strain. Mortality and BW were monitored for 2 wk postinfection. (*C*) Spike-specific IgG titers of K18-hACE2 mice surviving the lethal B.1.351 or B.1.617.2 infection. Dash denotes that mice died during infection.

We used the K18-hACE2 transgenic mouse model bearing human-like ACE2 to test in vivo efficacy of these nanobodies. The K18-hACE2 mice infected with B.1.351 and B.1.617.2 variants had scattered foci of inflammation in the lung, vasculitis, and neuronal degeneration, as revealed by hematoxylin and eosin (H&E) staining (Fig. 4*A*). The lethal infection of either B.1.351 or B.1.617.2 resulted in 100% mortality and up to 30% body weight (BW) loss in K18-hACE2 mice (Fig. 4 *B*, *Top*). Conversely, K18-hACE2 mice receiving 7A3 V_H_H-hFc fusion protein or the 2-in-1 mixture of 7A3 and 8A2 at a dose of 5 mg/kg had 100% survival after lethal B.1.351 infection, with no BW drop within the 2-wk observation period (Fig. 4 *B*, *Left*). However, two out of four mice receiving 5 mg/kg of 8A2 died within 8 d after infection (Fig. 4 *B*, *Left*). We did not measure pulmonary viral titers, due to the limited supply of the transgenic mice available in our study during the pandemic. Instead, we measured spike-specific IgG titers in the surviving mice. The surviving mice at 4 wk after lethal B1.351 infection had spike-specific IgG geometric mean titer (GMT) of 226 (2-in-1 mixture group), 3,620 (7A3 treatment), and 5,120 (8A2 treatment) (Fig. 4*C*). This indicated that the 2-in-1 mixture or 7A3 alone at a 5 mg/kg dose was more efficient than 8A2 alone to reduce viral load and protect K18-hACE2 mice from lethal B.1.351 infection. However, when K18-hACE2 mice were subjected to a lethal dose of B.1.617.2, the protective efficiency of the 2-in-1 mixture (5 mg/kg) was reduced to 50%, and the infected mice lost >20% of their initial BW (Fig. 4 *B*, *Right*). The microneutralization assay indicated that 7A3 exhibited higher IC_50_ against B.1.617.2 than B.1.351, while 8A2 completely lost neutralization activity against B.1.617.2 (Fig. 3*B*). Consistent with the microneutralization results, a dose of 10 mg/kg of 7A3 was required to achieve 75% protection in K18-hACE2 mice from B.1.617.2 lethality, whereas 8A2 was ineffective even at this higher dose (Fig. 4 *B*, *Right*). The K18-hACE2 mice that survived the lethal B.1.617.2 infection developed comparable spike-specific IgG titers in the 7A3 group (IgG GMT of 5120) and the 2-in-1 mixture group (IgG GMT of 3620) at 4 wk postadministration (Fig. 4*C*).

### Structure Complexes of the SARS-CoV-2 Spike and Dromedary Camel V_H_Hs.

**Fig. 5.**
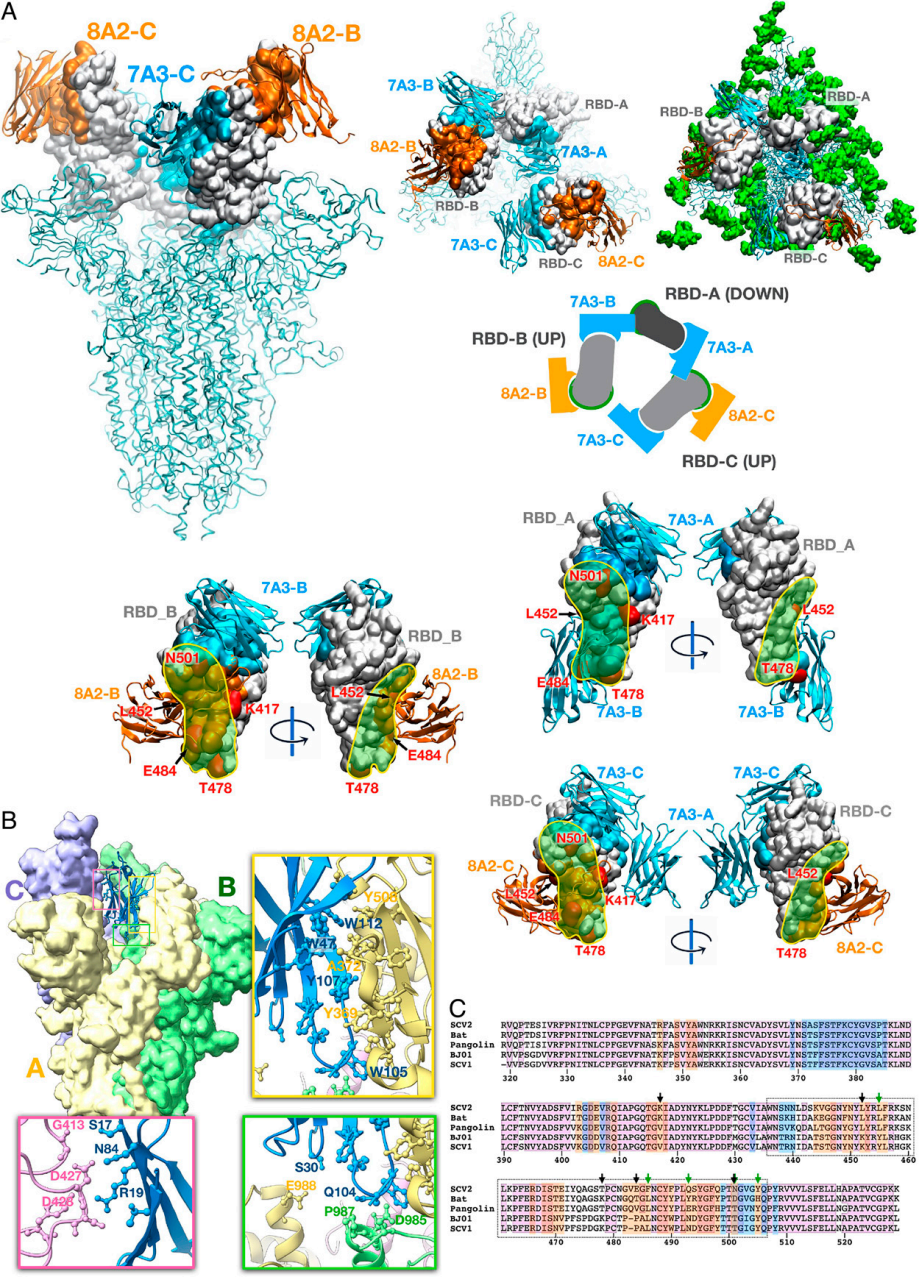
Cryo-EM structure of 7A3 and 8A2 nanobodies with SARS-Cov-2 spike. (*A*) The structure models based on the EM images show that 8A2 and 7A3 bind two distinct sites on the RBD. The mutations on the RBD derived from B.1.1.7 (N501Y), B.1.351 (K417N, E484K, N501Y), P.1 (K417T, E484K, N501Y), and B.1.617.2 (K417N, L452R, and T478K) are indicated. Green spheres (*Top Right*) are glycans. (*B*) The unique 7A3 binding pattern. (*C*) Nanobodies epitopes coverage as determined from the experimental structure using a 5-Å contact cutoff. Sequences of coronavirus RBD for Bat_RaTG13 (A0A6B9WHD3), Human BJ01 (Q6GYR1), Pangolin (A0A6G6A2Q2), SARS-CoV-2 (P0DTC2), and SARS-CoV-1 (P59594) are presented for comparison purposes. Degree of sequence identity is indicated by background pink hue. The 7A3 contact is indicated in blue, and 8A2 is indicated in orange. Arrows indicate mutation sites of concern (black) and key ACE2 contact residues (green). RBM is indicated by the dotted box. PDB entry codes for our complex structures of the 7A3 and 8A2 V_H_Hs with the SARS-CoV-2 stabilized spike: PDB ID 7TPR, EMD-26062.

To precisely characterize the binding of nanobodies on SARS-CoV-2 spike protein, we solved the cryo-EM structures of the SARS-CoV-2 spike in complex with the 8A2 and 7A3 dromedary camel V_H_Hs (*SI Appendix*, Table S4). The overall resolution estimates for the complexes were 3.4, 3.8, and 2.4 Å. The higher resolution for the cryo-EM complexes can be attributed to two major factors: the instrument used to collect a large dataset and the reduced movement of the RBD locked by two nanobodies as evidenced by the local-resolution map (*SI Appendix*, Fig. S9). Local refinement of the regions of interest on the complex involving the V_H_Hs and the RBD yielded three partial maps at overall resolutions of 3.4, 2.7, and 2.6 Å (*SI Appendix*, Figs. S9–S11) with statistical validation for the cryo-EM models (*SI Appendix*, Figs. S12 and S13). The final complex structure showed 8A2 V_H_H interacts with the epitope directly overlapping the site for ACE2 binding when the RBD is in the up mode (Fig. 5*A* and *SI Appendix*, Fig. S9). Interestingly, the 8A2 V_H_H has the most extended CDR3 loop (21 aa) that uniquely penetrates the N-terminal part of the RBD for direct binding of ACE2. The 8A2–RBD interaction showed minor variations in the two poses observed (8A2-RBD_B and 8A2-RBD_C), with a typical pattern involving sections from three β strands forming the V_H_H’s framework extending from Thr33-Ala40, Gly44-Trp47, Tyr95-Thr101, and Gln120. The 7A3 V_H_H binds an area distinct from the 8A2 site, as shown in its binding to RBD_B and RBD_C. The 7A3 V_H_H can bind the RBD in both up (RBD_B and RBD_C) and down (RBD_A) modes (*SI Appendix*, Figs. S9 and S11). The 7A3 V_H_H binds the RBD with its CDR3 (Tyr102 to Gly113) and an extended cluster of aromatic and hydrophobic residues including Phe37, Leu45, Trp47, Tyr59, Trp101, Tyr107, and Trp112. These residues form a well-structured motif interacting with RBD residues Tyr508, Phe374, Phe377, and Tyr369, and the carbonyl of Phe374 (Fig. 5 *B* and *C*). The RBD binding motif of the 7A3 V_H_H ends at the tip of CDR3 by Trp105 and is a common pattern to all three poses of 7A3. Although 7A3 can bridge two RBDs in up–down or down–up conformation, the up–up disposition does not allow 7A3 bridging. When the RBD is in its down mode (RBD_A), both 7A3-A and 7A3-B can bind the RBD-A, while 7A3-B interacts with the region partially overlapping the ACE2 binding site. This interaction does not interfere with 8A2 binding, only observed in the up conformation of the RBD. This observation is consistent with our biophysical cross-competition data on Octet, showing that 7A3 can also inhibit ACE2 binding to the RBD. Interestingly, the 7A3_A V_H_H forms interactions with both chain B and chain C of the spike (Fig. 5*B*). Ser17 and Asn84 of 7A3_A engage Gly413 and Asp427, respectively, on chain C. Furthermore, we observed that the interaction network formed at the tip of the 7A3_A V_H_H, buried deep in the structure of spike, where Trp105 and Gln104 of 7A3 engage Asp985, Glu988, and Pro987 in chain B of the spike.

Based on our cryo-EM complex structure using a 5-Å contact cutoff (Fig. 5*C*), 7A3 binds the regions (blue) with a majority of contact residues identical between SARS-CoV-2 (SCV2) and SARS-CoV (SCV1). The 8A2 binds the regions (orange), including the ACE2 binding motif (dotted box), but a majority of the 8A2 contact residues are not identical between SARS-CoV-2 and SARS-CoV; therefore, the binding of 8A2 to the RBD of SARS-CoV is weaker than that of 7A3 (Fig. 1*C*). Since 8A2 and 2F7 bind overlap epitopes (Fig. 1*E*), and their neutralizing activities against the VOCs are largely different (Figs. 2 and 3), we made an effort to pursue the 2F7 V_H_H/spike complex structure to compare it with 8A2 V_H_H/spike (*SI Appendix*, Fig. S14). Although the 2F7 map resolution is insufficient to resolve the features of the V_H_H, the map unambiguously identifies the general epitope area, confirming 2F7 shares the same binding area as 8A2. A refined structure of the 2F7 complex cannot be generated, due to the low resolution of the map. Based on our live virus data (Fig. 3 and *SI Appendix*, Table S3), 2F7 had reduced potency against B.1.1.7 and completely lost potency against B.1.351 and P.1. The 2F7 also lost binding against the RBD of B.1.617.2 (Fig. 1*D*). These data indicate that N501Y reduces the potency of 2F7, not 8A2, and that E484K and K417N/T in B.1.351 and P.1 completely compromised 2F7 activity, whereas they even increased the potency of 8A2. The 8A2 is more potent than 7A3 for the B.1.1.7, B.1.351, and P.1 variants, although 8A2 is less potent for the ancestral Wuhan-Hu-1 (Fig. 3 and *SI Appendix*, Table S3). The data indicate that the N501Y mutation in B.1.1.7 increases the potency of 8A2 and that E484K and K417N/K417T further increase the potency of 8A2. Our cryo-EM structure shows that the 8A2 V_H_H/RBD interaction can accommodate the K417 mutations. K417N/T could possibly be beneficial for 8A2 binding, due to the proximity of hydrophobic residues from 8A2 CDR3 (A112, F113), which may interact with the threonine methyl group, with the −OH moiety remaining solvent accessible. E484K is an 8A2 contact residue (Fig. 5*C*). E484K may increase 8A2 affinity by facilitating favorable interactions with Y95, Y115, or Y117 of the V_H_H. The exact nature of the interaction is difficult to assign, due to the flexibility of the RBD loop 456 through 491. Leu452, another interacting residue with the 8A2 V_H_H (Fig. 5*C*), appeared critical for nanobody binding, because the mutation of this residue in the B.1.617.2 spike might contribute to the loss of 8A2 binding (Fig. 1*D*). It is interesting to note that the two extra cysteine residues in V_H_H 8A2, one (Cys32) located in CDR1 and the other (Cys108) in CDR3, do not form a disulfide bond, based on their distance (>20 Å) in our cryo-EM complex structure. Previous studies, by substituting the extra cysteines, showed V_H_Hs could function without a noncanonical disulfide bond (20, 21). In some cases, the affinity was not reduced, indicating the extra disulfide bond might have evolved to stabilize the biophysical properties of camel V_H_Hs, rather than the affinity and specificity of antigen binding (23). Furthermore, our findings show that both nanobodies were inserted into the relatively glycan-free regions of the RBD (Fig. 5 *A*, *Top Right*). Taken together, our structure analysis based on cryo-EM maps supports the finding that the 8A2 and 7A3 V_H_Hs bind two distinct sites on the RBD. Whereas 8A2 disrupts the ACE2 binding with a long CDR3 of the dromedary camel nanobody that penetrates the N-terminal part of the RBD, we found that 7A3 binds a unique buried site that involves the residues of the S2 subunit of the spike, independent of the conformational state of the RBD.

## Discussion

In the present study, we constructed sizeable V_H_H phage libraries from six dromedary camels and isolated potent neutralizing nanobodies 7A3 and 8A2 against SARS-CoV-2. The 7A3 recognizes a previously undescribed and deeply buried site containing residues from both the S1 and S2 subunits in the SARS-CoV-2 spike protein. This epitope is unique for the 7A3 nanobody, possibly underlying its broad neutralization activity against various variants both in vitro and in vivo, including mice infected with the B.1.617.2 variant. The 8A2 V_H_H binds the spike protein with a long CDR3 that directly interferes with ACE2 binding when the RBD is in its up mode, whereas the 7A3 nanobody binds the RBD in both up and down modes. Consequently, mixture treatments of nanobodies 7A3 and 8A2 have potent neutralization activities against multiple SARS-CoV-2 variants. In addition, the 7A3 nanobody alone protects mice from B.1.617.2 variant infection. The critical contribution and finding of this study are our creation of large dromedary camel V_H_H phage libraries and using the unique camel libraries for isolation of nanobodies that have an affinity (*K*_D_ < 1 nM) without a need for affinity maturation and exhibit broad neutralizing activities against SARS-CoV-2 and its variants. This work suggests that our dromedary camel libraries could be useful to isolate neutralizing nanobodies against future emerging viruses.

Nanobodies are small, stable, and easy to produce (24). As a result, several nanobodies have been produced during the pandemic to serve as alternates to conventional antibodies (25). The two major nanobodies, H11-H4 and VHH72, have been isolated. H11-H4 was isolated from a naive llama V_H_H library by phage display against the RBD of SARS-CoV-2. The best V_H_H binder has a low affinity (*K*_D_ < 1 μM). After affinity maturation, the final V_H_H binder has an estimated *K*_D_ of 5 nM to 10 nM. H11-H4 can interfere with ACE2 binding with a small overlap with the ACE2 contact surface. The 8A2 interferes with ACE2 binding with an unusual long CDR3 that penetrates the N-terminal part of the RBD and interacts with all the critical residues for ACE2 binding (Fig. 5*C*). Overall, 8A2 has a higher affinity (*K*_D_ < 1 nM) and broader coverage of the ACE2 binding residues than H11-H4. While their IC_50_ values (low nanomolars) are similar on the ancestral Wuhan-Hu-1 live virus, unlike 8A2, H11-H4 has not been tested on emerging VOCs. Another nanobody called VHH72 was isolated by panning a phage library made from a llama immunized by SARS-CoV and MERS-CoV spike proteins (26). However, the affinity for SARS-CoV-2 RBD is also low (*K*_D_ ≈ 40 nM). VHH72 has been tested only on pseudovirus. It has not been tested on the live virus of SARS-CoV-2 and its emerging variants. Like VHH72, 7A3 binds the spike proteins of both SARS-CoV-2 and SARS-CoV. The crystal structure of VHH72 and the SARS-CoV-1 RBD has been solved. It is not clear how VHH72 would interact with the spike trimer protein of SARS-CoV-2. Interestingly, our 7A3 binds a deeply buried site in the spike that engages the apex of the S2 subunit. Based on the limited structure data about VHH72 on the RBD, the epitope of 7A3 does not overlap with that of VHH72. The 7A3 can bind the RBD independent of its up or down mode. We found that 7A3 V_H_Hs could contribute to the stabilization of the two up arms by bridging the down and up conformations. The unique 7A3 binding pattern might be necessary for the nanobody to achieve its maximum inhibitory effect on ACE2. The Asp985–Glu988 interaction seems highly conserved (Fig. 5*B*), indicative of a conserved function and lack of immune surveillance due to the buried disposition of the region. This arrangement suggests a novel mechanism for stabilizing the spike by 7A3. This peculiar arrangement requires a spike disposition with one RBD down, which may inhibit the full engagement of the spike and serve as a kinetic hindrance. Pro987, a part of the 7A3 epitope, is one of the stabilizing substitutions used to keep the spike in the prefusion conformation. Position 987 is lysine in wild-type spikes. Our functional data show that 7A3, which was panned on the stabilized spike with K986P and V987P mutations, can neutralize the wild-type and variants of SARS-CoV-2 live viruses without the K986P and V987P substitutions. The combination of 7A3 with 8A2 may provide a novel mechanism to maximize the intervention’s efficacy by addressing the spike’s stability (potentially affecting the kinetics of the spike rearrangement) while targeting a low mutability spot in the spike, and blocking ACE2 binding.

In the K18-hACE2 mouse model, B.1.351 is 100 times more lethal than the original Wuhan-Hu-1 strain (27). In our study, although 8A2 alone was more potent than 7A3 alone against the B.1.351 variant in cell-based neutralization assays, 7A3 exhibited higher efficacy in B.1.351-challenged mice, suggesting 7A3 and 8A2 might have different organ absorption, distribution, metabolism, and excretion properties in living organisms. K18-hACE2 mice receiving the 2-in-1 mixture of 8A2 + 7A3 nanobodies at 5 mg/kg were protected entirely and exhibited no sickness and no BW loss following a lethal challenge of B.1.351. However, the mixture of 8A2 + 7A3 nanobodies at 5 mg/kg only achieved 50% protection in mice challenged with B.1.617.2. The cell-based microneutralization assay showed that the B.1.617.2 variant completely escaped the neutralization by 8A2 alone, while the variant remained susceptible to 7A3. Consistently, 7A3 alone at 10 mg/kg outperformed the mixture of 8A2 + 7A3 by achieving 75% protection against the lethal B.1.617.2 challenge in mice. Investigating the pharmacokinetics of 7A3 alone or the 2-in-1 mixture with 8A2 would be needed for clinical development. Nevertheless, all surviving mice developed an antiviral spike humoral response, which may provide long-term protection in the host. This finding is highly interesting because our data might suggest that neutralizing nanobodies such as 7A3 and 8A2 would not prevent the host from developing their immune response against the viral infection.

This work establishes large camel V_H_H phage libraries that produce high affinity and broad neutralizing agents against the emerging viral pandemic. The approach may be useful in identifying unique epitopes as compared to those conventional antibodies identified by human B cell cloning and animal immunization approaches. The two nanobodies (7A3 and 8A2) reported here have therapeutic potential as passive immunotherapy and valuable building blocks for developing multidomain (28) and multispecific drugs for the challenge posed by current and future emerging variants. The nanobodies can also be used as inhaled drugs for treating COVID-19 and other respiratory diseases (29). The characterization of the unique 7A3 epitope structure might provide insights for vaccine design broadly targeting SARS-CoV-2 variants and similar coronaviruses in the future.

## Materials and Methods

### Reagents Related to COVID-19.

The following reagents were obtained through BEI Resources, National Institute of Allergy and Infectious Diseases (NIAID), NIH, SARS-Related Coronavirus 2: 1) isolate USA-WA1/2020 bearing 614D, NR-52281 (deposited by the Centers for Disease Control and Prevention); 2) isolate New York-PV09158/2020 bearing 614G, NR-53516; 3) isolate USA/CA_CDC_5574/2020 (B.1.1.7 or Alpha), NR-54011 (deposited by the Centers for Disease Control and Prevention); 4) isolate hCoV-19/South Africa/KRISP-EC-K005321/2020 (B.1.351 or Beta), NR-54008 contributed by Alex Sigal and Tulio de Oliveira, University of KwaZulu-Natal, Durban, South Africa; 5) isolate hCoV-19/Japan/TY7-503/2021 (Brazil P.1 or Gamma), NR-54982; 6) Spike Glycoprotein (Stabilized) from SARS-Related Coronavirus 2, Wuhan-Hu-1 with C-Terminal Histidine Tag, Recombinant from Baculovirus, NR-52396; 7) Vector pCAGGS containing the SARS-Related Coronavirus 2, Wuhan-Hu-1 (or USA-WA-1) Spike Glycoprotein Receptor Binding Domain (RBD), NR-52309; and 8) isolate B.1.617.2 (Delta) Lot 3002648422 provided by Bin Zhou, the Centers for Disease Control and Prevention, Atlanta, GA. All live viruses were amplified in Vero E6 (ATCC, CRL-1586) or TMPRSS2-E6 (BPS Bioscience, #78081), and aliquots were stored at −80 °C until use.

### Construction of Dromedary Camel V_H_H Phage Libraries.

The construction of the camel library followed our previous protocol (30) for the construction of a shark V_NAR_ phage library with some modifications. The PCR was performed with the forward primer CALL001 (5-GTCCTGGCTGCTCTTCTACAAGG-3′) and reverse primer CALL002 (5-GGTACGTGCTGTTGAACTGTTCC-3′). Six forward primers (CamelS1-F through CamelS6-F) were designed according to the IGHV1 genes of the Arabian camel (*C. dromedarius*) (https://www.imgt.org/IMGTrepertoire/). The V_H_H reverse primer (CamelVHH-R) was synthesized based on the framework four (FR4) regions of V_H_H. The amplified V_H_H fragments were assembled with vector backbone by EASeL, an overlap extension PCR method for library construction (30). The phagemid vector backbone pComb3X fragment was prepared by PCR using the Vector-F and Vector-R primers and Phusion Hot Start II High-Fidelity DNA Polymerase (Thermo Scientific). Six camel V_H_H genes were assembled with the pComb3X phagemid vector backbone using the F-Linker and Vector-R primers by EASeL (*SI Appendix*, Table S5). For library construction, the purified overlapped PCR products were self-ligated and transformed into TG1 electrocompetent cells (Lucigen). The size of each camel V_H_H phage library in terms of colonies is ∼6 × 10^10^. The total diversity of six dromedary camel V_H_H libraries used in the present study is ∼4 × 10^11^. The next-generation sequencing was used to validate the library diversity as we did previously with our shark V_NAR_ phage library (30).

### Phage Panning and ELISA.

Please refer to previous publications for phage panning protocols (31, 32). In one approach, Maxisorp immune tubes (Thermo Scientific) were coated with SARS-CoV-2 RBD (Sino Biological, 40592-V08B) for the first and second round of panning and were coated with SARS-CoV-2 S stabilized trimer (BEI, NR52396) for the third and fourth rounds of panning. In another approach, SARS-CoV-2 RBD was used for three rounds of panning and switched to SARS-CoV-2 S trimer for the fourth round. The S protein trimer that was used in our phage panning is modified to remove the polybasic S1/S2 cleavage site (RRAR to A; residues 682 to 685), stabilized with a pair of mutations (K986P and V987P) and includes a thrombin cleavage site, T4 foldon trimerization domain, and C-terminal hexahistidine tag.

After phage panning, single colonies were picked for monoclonal ELISA. Maxisorp 96-well plates (Fisher Scientific, 12565136) were coated with SARS-CoV-2 RBD, S trimer, or SARS-CoV-1. Phage ELISA was performed following previous protocols (31, 32), and absorbance was read using a spectrophotometer (Molecular Devices) at 450 nm.

### Analysis of Complementarity-Determining Regions of Dromedary Camel V_H_Hs.

Sequence alignment of SARS-CoV-2 neutralizing V_H_Hs using Clustal Omega Program (https://www.ebi.ac.uk/Tools/msa/clustalo/) with ImMunoGeneTics (IMGT) in bold, Kabat italicized, and Paratome underlined. IMGT and Kabat were determined using IgG Blast (https://www.ncbi.nlm.nih.gov/igblast/), and Paratome was determined using Ofran Lab service (https://ofranservices.biu.ac.il/site/services/paratome/).

### Dromedary Camel V_H_H Protein Production.

Nanobodies (V_H_H-His) were produced in *E. coli* as previously described (30). Briefly, the phagemids containing the V_H_H binders were transformed into HB2151 *E. coli* strain. The nanobody colonies were grown in 2 L of 2YT media containing 2% glucose, 100 μg/mL ampicillin at 37 °C, until the optical density at 600 nm (OD_600_) reached 0.8 to 1. Culture media was then replaced with 2YT media containing 1 mM IPTG (Sigma), 100 μg/mL ampicillin, and shaken at 30 °C overnight for soluble protein production. The bacteria pellet was spun down and lysed with polymyxin B (Sigma) for 1 h at 37 °C to release the soluble protein. The supernatant was harvested after lysis and purified using the HisTrap column (GE Healthcare) on AKTA Explorer (GE Healthcare). Nanobody production yields vary in *E. coli*. The yield of 7A3 V_H_H and 8A2 V_H_H in *E. coli* is 44 mg/L and 6 mg/L with over 95% purity on sodium dodecyl sulfate polyacrylamide gel electrophoresis.

Nanobodies are also produced in Fc fusions as previously described (33). Briefly, the V_H_H sequence was fused with human IgGγ1 Fc. The final plasmid was transfected into Expi293 cells (ThermoFisher), and the protein was purified using the protein A column (GE healthcare).

### Affinity Measurement and Competition Assay by Octet.

The binding kinetics and competition assay were determined using the Octet RED96 system (FortéBio) at the Biophysics core at National Heart, Lung, and Blood Institute (NHLBI), NIH. For binding kinetics, the SARS-CoV-2 original or mutant variants were immobilized onto Ni–nitriloacetic acid (NTA) sensor tips (Fortébio). The antigen-coated tips were then dipped into phosphate-buffered saline (PBS) to stabilize the curve, transferred into 25 nM V_H_H-hFc for the association, and, finally, dipped into PBS for dissociation. Raw data were processed using Octet Data Analysis Software 9.0 (FortéBio) to determine the K_D_ value using a 1:1 binding model.

For the competition assay, the SARS-CoV-2 RBD-His was immobilized onto Ni-NTA sensor tips. The resulting RBD-coated tips were then dipped into either PBS or 500 nM first nanobodies. After loading, the sensor tips were briefly incubated in PBS before being dipped into wells containing 500 nM competing nanobody, followed by a dissociation step in PBS. Raw data were processed using Octet Data Analysis Software 9.0. Residual binding was calculated as follows: (response signal from the second ligand in the presence of first ligand/response signal from the second ligand in the absence of the first ligand) × 100.

### Epitope Mapping by RBD Peptide Arrays.

To map the V_H_H binding epitopes, we produced a set of overlapping synthetic peptides based on the SARS-CoV-2 RBD sequence followed by ELISA. Briefly, we designed a total of 24 peptides of 18 amino acids in length with nine overlapping residues (Genscript). The 24 peptides were coated on a Maxisorp 96-well plate (Fisher Scientific, 12565136). Five μg/mL anti-RBD V_H_Hs-hFc was added to the plate, followed by horseradish peroxidase (HRP)-conjugated anti-human IgG antibody (Jackson ImmunoResearch). The binding activity was determined using a spectrophotometer (Molecular Devices) with the absorbance read at 450 nm. Experiments were performed in triplicate and repeated three times with similar results.

### ACE2 Inhibition Assay.

A Ni-NTA ELISA plate (Thermo Scientific) was coated with ACE2-His. V_H_H-hFc was incubated with varying concentrations of RBD-mFc starting from 1 μg/mL with 1:3 dilutions. The V_H_H-hFc and RBD-mFc mixture was then added to the ACE2-His–coated plate, and binding was detected using a goat anti-mouse Fc HRP conjugate (Jackson ImmunoResearch). Absorbance at 450 nm was read using a spectrophotometer (Molecular Devices).

### Flow Cytometry.

The spike coding sequences for SARS-CoV Urbani and SARS-CoV-2 in pcDNA3.1 (+) plasmid were kindly provided by Alex Compton, National Cancer Institute (NCI), Frederick, MD. These sequences were then codon optimized for human cell expression, followed by a 5′ Kozak expression sequence (GCCACC), a 3′ tetra-glycine linker, and a FLAG-tag (DYKDDDDK) (34). A431, a human epidermoid carcinoma cell line, was transfected with either pcDNA3.1 (+)-SARS-CoV-spike vector or pcDNA3.1 (+)-SARS-CoV2-spike vector by either FuGENE HD (Promega) or Lipofectamine 3000 (ThermoFisher). The A431 clones with high viral spike protein expression on the cell surface were sorted by flow cytometry using the control CR3022 antibody for SARS-CoV and SARS-CoV-2 (SinoBiological). The A431-CoV-2-S or A431-CoV-S cell lines were stained with camel V_H_H-hFc followed by goat anti-human IgG-APC (Jackson ImmunoResearch). Data were collected using the BD FACSCanto II Cell Analyzer.

### Pseudoviral Neutralization Assays.

Three pseudoviral neutralization assays were conducted independently in different laboratories to validate the antiviral activity of the camel V_H_H nanobodies. In the first assay, called lentivirus-based pseudovirus infection assay, HEK293T cells expressing human ACE2 (HEK293T-ACE2) (kindly provided by Nicole Doria-Rose and Kizzmekia S. Corbett, Vaccine Research Center, NIAID, Bethesda, MD) were seeded in 96-well plates. The appropriate volume of SARS-CoV-2 or SARS-CoV spike pseudovirus supernatant was used to produce a luciferase signal 1,000× higher than the baseline. V_H_H-hFc fusion proteins were prepared in 12-point twofold serial dilutions starting with 50 μg/mL for SARS-CoV-2. For the “2-in-1 cocktail,” V_H_H-hFcs were prepared in 12-point twofold serial dilutions starting with 25 μg/mL of each nanobody for a total of 50 μg/mL. Nanobodies and viruses were mixed for 45 min before adding to HEK 293T-hACE2 cells. After incubation for 72 h, the luciferase signal was measured by the plate reader. All experiments were performed in triplicate. The infectivity was calculated from the luciferase activity of different groups normalized to the virus-only group (100%). The IC_50_s and IC_90_s were calculated with GraphPad Prism using nonlinear fit for log(inhibitor) vs. normalized response − variable slope.

In the pseudotyped particle (PP) entry assay, SARS-CoV-2-S Wuhan-Hu-1, B.1.351, P.1, and B.1.1.7 PPs were purchased from Codex Biosolutions and were produced using a murine leukemia virus pseudotyping system (35). The variant spike sequences clones were B.1.1.7 (del69-70, del144, N501Y, A570D, D614G, P681H, T716I, S982A, and D1118H), B.1.351 (L18F, D80A, D215G, del242-244, K417N, E484K, N501Y, D614G, and A701V), and P.1 (L18F, T20N, P26S, D138Y, R190S, K417T, E484K, N501Y, D614G, H655Y, T1027I, V1176F). Expi293F cells with stable expression of human ACE2 (HEK293-ACE2) were generated at Codex BioSolutions. For the PP entry assay, HEK293-ACE2 cells were seeded in 384-well microplates (Greiner BioOne). The cells were incubated at 37 °C with 5% CO_2_ overnight. V_H_H-hFc fusion proteins at 120-μg/mL were titrated 1:3 in Dulbecco's PBS for 12 concentration points and added to cells in triplicates. Cells were incubated with nanobodies for 1 h at 37 °C, 5% CO_2_, before SARS-CoV-2-S PP was added. The plates were then spinoculated by centrifugation at 1,500 rpm (453 × *g*) for 45 min and incubated at 37 °C for 48 h to allow cell entry of PP and expression of the luciferase reporter. After the incubation, the supernatant was removed with gentle centrifugation using a Blue Washer (BlueCat Bio). Then Bright-Glo Luciferase detection reagent (Promega) was added to assay plates and incubated for 5 min at room temperature. The luminescence signal was measured using a PHERAStar plate reader (BMG Labtech). Data were normalized with wells containing PPs as 100% and wells containing no PP (media control) as 0%. All nanobodies were also assessed for cytotoxicity as a counterassay using the same cell treatment and incubation protocol, omitting the PP addition, and assaying for adenosine 5′-triphosphate content with an ATPLite (PerkinElmer) cytotoxicity kit.

The third assay is called the pseudovirus fluorescence reporter assay (36). HEK-293T cells expressing human ACE2 were plated at a density of 50,000 cells per well in a six-well plate. Cells were transduced with SARS-CoV-2 pseudotyped lentiviruses expressing red fluorescence protein (S-CD512-EF1a-RFP) with a multiplicity of infection (MOI) of 0.5± nanobodies at various concentrations. Forty-eight hours posttransduction, cells were harvested and fixed in 1% formaldehyde. A BD LSRFortess Flow Cytometer was used to determine percent fluorescent cells and the mean fluorescent intensity per sample. All experiments were performed in triplicate.

### Live SARS-CoV-2 Neutralization Assay.

Two live virus assays were conducted independently in different laboratories. The first assay is called SARS-CoV-2 CPE assay. The CPE assay was performed at the Southern Research Institute (37). Briefly, V_H_H-hFc fusion proteins were titrated in PBS and acoustically dispensed into 384-well assay plates at 600 nL per well. Next, cell culture media (minimum essential media, 1% Pen/Strep/GlutaMax, 1% Hepes, 2% HI fetal bovine serum) was dispensed at 5 μL per well into assay plates and incubated at room temperature. Vero E6, previously selected for high ACE2 expression, was dispensed to the plate at 4,000 cells per well in 10 μL of media. The cells were incubated with nanobodies for 30 min before SARS-CoV-2 (USA_WA1/2020) was inoculated at 0.002 M.O.I. in 15 μL per well media. Assay plates were incubated for 72 h at 37 °C, 5% CO_2_, 90% humidity. Then, CellTiter-Glo (Promega) was dispensed and incubated for 10 min at room temperature, and luminescence signal was read on an EnVision plate reader (PerkinElmer). Data were normalized with wells containing the virus as 0% CPE rescue and wells without virus (media control) as 100% CPE rescue.

The second assay was a live SARS-CoV-2–based microneutralization assay. Virus titers were determined using an ELISA-based 50% tissue culture infectious dose (TCID_50_) method (27). Vero E6 cells were preseeded in 96-well tissue culture plates overnight. The next day, individual V_H_H-hFc fusion proteins or 2-in-1 mixtures were serially diluted, and were incubated with 10^2^ TCID_50_ of the live virus at room temperature for 1 h. The virus–nanobody mixtures were then added to Vero E6 cells preseeded in 96-well flat-bottom tissue culture plates and incubated at 37 °C, 5% CO_2_ for another 48 h. The residual virus was detected using in-house–developed SARS-CoV-2–specific rabbit polyclonal antibodies (27) followed by goat anti-rabbit IgG with HRP (Invitrogen). All V_H_H-hFc fusion proteins were tested at the starting concentration of 120 or 60 nM in the 2-in-1 mixtures.

### Animal Testing.

All procedures were performed according to the animal study protocols approved by the Food and Drug Administration (FDA) White Oak Animal Program Animal Care and Use Committee. Hemizygous 2B6.Cg-Tg(K18-ACE2)2Prlmn/J (K18-hACE2) transgenic mice (JAX) were bred in the FDA White Oak vivarium and were genotype confirmed before the experiments. All subsequent live virus infection experiments were conducted in the FDA animal biosafety level 3 laboratory.

K18-hACE2 mice infected with the hCoV-19/South Africa/KRISP-EC-K005321/2020 (B.1.351, Beta) or B.1.617.2 (Delta) variant were humanely killed after they reached the moribundity on day 6 or 7 postinfection. Lung and brain tissues were harvested and fixed in 10% neutral buffered paraformaldehyde for at least 2 wk before histology. Fixed lung and brain tissues were embedded in paraffin and were sectioned followed by the standard H&E staining (Histoserv). Uninfected mouse lung and brain were used as a negative control. H&E images were captured using Leica Aperio AT2 slide scanner (Histoserv) and viewed using Aperio ImageScope DX clinical viewing software (version 12.4.3.5008). Adult K18-hACE2 mice were injected with individual V_H_H-hFc fusion proteins (7A3 and 8A2) at 5 or 10 mg/kg or the 2-in-1 mixture at 5 mg/kg via the intraperitoneal route. Approximately 2 h later, mice were anesthetized under isoflurane and were intranasally inoculated with B.1.351 or B.1.617.2 at 10^2^ TCID_50_ per mouse. BW and mortality were monitored daily for up to 14 d postinfection. All efforts were made to minimize animal suffering, and mice reaching predefined humane endpoints (e.g., 30% BW loss) were immediately killed.

The mice that survived the infection were tail bled at approximately 4 wk postinfection, and sera were collected for spike-specific IgG ELISA as described before (27). All sera were heat inactivated at 56 °C for 30 min before ELISA. Mouse sera were serially diluted in PBS (pH 7.4) and added to recombinant spike precoated 96-well microtiter plates (27). Bound mouse IgG was probed using peroxidase-conjugated goat anti-mouse IgG (Invitrogen) followed by One-step TMB substrate (ThermoFisher). The endpoint IgG titers were the reciprocals of the highest serum dilutions that yielded more than twofold the OD of that of PBS blank at 450 nm.

### Cryo-EM Specimen Preparation.

The SARS-CoV-2 (Wuhan) stabilized spike protein ectodomain with six proline substitutions was kindly provided by Dominic Esposito at NCI, Frederick, MD (*SI Appendix*, Fig. S15) (38). Complexes of spike with 7A3 and/or 8A2 V_H_Hs were prepared by mixing the components at a spike trimer to nanobody molar ratio of 1:6. The final concentration of spike trimer was 3 μM in PBS at pH 7 with the addition of 5 mM imidazole. Because the 8A2 stock was too diluted, complexes involving this nanobody were prepared at 0.5 μM spike trimer followed by a sixfold concentration using a 10-kDa cutoff centrifugal filter (Amicon Ultra). All complexes were incubated on ice for at least 5 min before grid preparation.

Specimens were plunge frozen on customized support grids consisting of C-flat R 1.2/1.3 (Protochips) supplemented with a 30-nm-thick gold layer applied on the grid bar side using a sputterer (Leica ACE-600). Prior to specimen deposition, grids were pretreated on a plasma cleaner (Tergeo) in immersion mode with a power of 38 W for a period of 75 s. A 3-μL aliquot of the prepared complex was laid on the surface of the grid inside the chamber of a vitrification robot (Leica EM-GP2) held at 22 °C with an relative humidity of 98%, blotted for 4 s using two layers of filter paper (Whatman Grade 1), immediately plunged into liquid ethane kept at 90 K, and transferred to liquid nitrogen for storage.

### Cryo-EM Data Collection and Image Processing.

Specimens were imaged on either a Thermo Fisher Scientific/Field Electron and Ion Company Talos Arctica operated at 200 KeV or a TFS/FEI Titan Krios operated at 300 KeV furnished with a Gatan image filter (GIF) operated in zero-loss mode with a slit of 20 eV. Micrographs were recorded as movies (*SI Appendix*, Table S5) on a Gatan K2-Summit or a K3 direct electron detector. Data preprocessing was performed in the context of Scipion 3. Movies were aligned using MotionCorr2 before contrast transfer function (CTF) determination using CTFFIND4.1. Motion-corrected dose-weighted micrographs were imported into Cryosparc for further processing. Individual molecular images were detected using the Topaz Extract machine learning algorithm. Extracted particles were curated utilizing a combination of two-dimensional classification rounds and ab initio refinement. “Clean” particles were then refined using a series of heterogeneous, unsymmetrized homogeneous, C3 symmetrized homogeneous, global CTF, and nonuniform refinements.

### Molecular Modeling.

The 8A2 and 7A3 models were obtained using homology modeling tools. The initial model of 8A2 revealed an unusually extended CDR3, so we challenged our model to ensure the quality of our initial guess. Yet Another Scientific Artificial Reality Application (YASARA) (39), Iterative Threading Assembly Refinement (I-TASSER) (40), and Rosetta (41) homology modeling packages were used for this purpose. Local installation of Rosetta (Linux version 2020 08.61146 bundle), I-TASSER (version 5.1), and YASARA (Mac version 20.10.4) were used. Standard scripts and parameters were used for Rosetta and I-TASSER. A YASARA homology modeling macro (HM_build) was used, allowing 25 templates, 10 alternative sequence alignments, and 50 loops per model. Finally, the structures obtained from all the modeling engines were combined using the YASARA HM_build macro to form a hybrid model. The hybrid model was allowed to be further refined against all templates using Feedback Restrain Molecular Dynamics (42, 43), resulting in a slight improvement of the Z score from 0.5 to 0.45 for 8A2 and 0.6 to 0.4 for 7A3, but showing a general agreement across the procedures used, which confirmed the unusual 8A2 CDR3 geometry. The nanobody models were subjected to molecular dynamics calculations [Gromacs 2021 (44)] to build a diverse set of conformations for macromolecular docking [ZDock v 3.0.2 (45)] and rigid body fitting to the maps using Chimera, version 1.15 (Mac build 42258) (46). Multiple orientations were obtained due to the lack of resolution of the initial map. We clustered the poses obtained, conducted molecular dynamics simulations starting from a set of hand-curated 240 most promising orientations, made Gromacs-2018-densfit (47) on an initial model obtained by fitting Protein Data Bank (PDB) ID 6x2B (45) to the density using Chimera (48), and aligned the N-terminal domain structure PDB ID 5x4S (49) and RBD structure PDB ID 7EAM. We then processed using a YASARA homology modeling macro with default parameters to generate missing loops and rigid body aligned to the corresponding sites in the spike reference structure (PDB ID 6x2B), followed by local fitting to density using Chimera. Molecular dynamics trajectories (44) were used to generate an epitope map based on the mean contact time of the nanobody with the RBD residues, weighted by the correlation coefficient reported by Gromacs-densfit using a 5-Å cutoff distance. Final structure refinement was performed using Phenix (1.19.2_4158) (50) followed by manual correction using Chimera. A model including glycosylation sites was generated following the PDB ID 6x2B glycosylated structure available in the Chemistry at Harvard Macromolecular Mechanics-graphical user interface (CHARMM-GUI) repository (https://www.charmm-gui.org/docs/archive/covid19). We also obtained the 2F7/spike map. A refined structure of the 2F7 complex was not generated, due to the low resolution of the map. The 2F7 pose was generated by manually correcting the overall fitting of a 2F7 model to the map using the program Chimera, version 1.14, with a homologous model of 2F7 generated from the coordinates of 8A2 (*SI Appendix*, Fig. S14).

## Supplementary Material

Supplementary File

## Data Availability

The data for cryo-EM structures have been deposited in the PDB under PDB ID 7TPR, and in the Electron Microscopy Databank under ID EMD-26062. Requests for further information and reagents should be directed to and will be fulfilled by the lead corresponding author, M.H. (homi@mail.nih.gov). All other data are available in the main text or *SI Appendix*.

## References

[1] Q. Li , Early transmission dynamics in Wuhan, China, of novel coronavirus-infected pneumonia. N. Engl. J. Med. 382, 1199–1207 (2020). 31995857 10.1056/NEJMoa2001316 PMC7121484

[2] F. Zhou , Clinical course and risk factors for mortality of adult inpatients with COVID-19 in Wuhan, China: A retrospective cohort study. Lancet 395, 1054–1062 (2020). 32171076 10.1016/S0140-6736(20)30566-3 PMC7270627

[3] M. Vaduganathan , Renin-angiotensin-aldosterone system inhibitors in patients with Covid-19. N. Engl. J. Med. 382, 1653–1659 (2020). 32227760 10.1056/NEJMsr2005760 PMC7121452

[4] R. Yan , Structural basis for the recognition of SARS-CoV-2 by full-length human ACE2. Science 367, 1444–1448 (2020). 32132184 10.1126/science.abb2762 PMC7164635

[5] M. Yuan , A highly conserved cryptic epitope in the receptor binding domains of SARS-CoV-2 and SARS-CoV. Science 368, 630–633 (2020). 32245784 10.1126/science.abb7269 PMC7164391

[6] J. Hansen , Studies in humanized mice and convalescent humans yield a SARS-CoV-2 antibody cocktail. Science 369, 1010–1014 (2020). 32540901 10.1126/science.abd0827 PMC7299284

[7] A. Baum , Antibody cocktail to SARS-CoV-2 spike protein prevents rapid mutational escape seen with individual antibodies. Science 369, 1014–1018 (2020). 32540904 10.1126/science.abd0831 PMC7299283

[8] M. Ho, Perspectives on the development of neutralizing antibodies against SARS-CoV-2. Antib. Ther. 3, 109–114 (2020). 32566896 10.1093/abt/tbaa009 PMC7291920

[9] L. Yang , COVID-19 antibody therapeutics tracker: A global online database of antibody therapeutics for the prevention and treatment of COVID-19. Antib. Ther. 3, 205–212 (2020). 33215063 10.1093/abt/tbaa020 PMC7454247

[10] L. R. Baden ; COVE Study Group, Efficacy and safety of the mRNA-1273 SARS-CoV-2 vaccine. N. Engl. J. Med. 384, 403–416 (2021). 33378609 10.1056/NEJMoa2035389 PMC7787219

[11] F. P. Polack ; C4591001 Clinical Trial Group, Safety and efficacy of the BNT162b2 mRNA Covid-19 vaccine. N. Engl. J. Med. 383, 2603–2615 (2020). 33301246 10.1056/NEJMoa2034577 PMC7745181

[12] M. J. Mulligan , Phase I/II study of COVID-19 RNA vaccine BNT162b1 in adults. Nature 586, 589–593 (2020). 32785213 10.1038/s41586-020-2639-4

[13] M. Hoffmann , SARS-CoV-2 variants B.1.351 and P.1 escape from neutralizing antibodies. Cell 184, 2384–2393 (2021). 33794143 10.1016/j.cell.2021.03.036 PMC7980144

[14] K. Wu , Serum neutralizing activity elicited by mRNA-1273 vaccine. N. Engl. J. Med. 384, 1468–1470 (2021). 33730471 10.1056/NEJMc2102179 PMC8008744

[15] J. Lopez Bernal , Effectiveness of Covid-19 vaccines against the B.1.617.2 (Delta) variant. N. Engl. J. Med. 385, 585–594 (2021). 34289274 10.1056/NEJMoa2108891 PMC8314739

[16] E. De Genst , Molecular basis for the preferential cleft recognition by dromedary heavy-chain antibodies. Proc. Natl. Acad. Sci. U.S.A. 103, 4586–4591 (2006). 16537393 10.1073/pnas.0505379103 PMC1450215

[17] E. I. Azhar , Evidence for camel-to-human transmission of MERS coronavirus. N. Engl. J. Med. 370, 2499–2505 (2014). 24896817 10.1056/NEJMoa1401505

[18] D. Wrapp , Cryo-EM structure of the 2019-nCoV spike in the prefusion conformation. Science 367, 1260–1263 (2020). 32075877 10.1126/science.abb2507 PMC7164637

[19] H. English, J. Hong, M. Ho, Ancient species offers contemporary therapeutics: An update on shark V_NAR_ single domain antibody sequences, phage libraries and potential clinical applications. Antib. Ther. 3, 1–9 (2020). 32118195 10.1093/abt/tbaa001 PMC7034638

[20] M. N. Mendoza, M. Jian, M. T. King, C. L. Brooks, Role of a noncanonical disulfide bond in the stability, affinity, and flexibility of a VHH specific for the Listeria virulence factor InlB. Protein Sci. 29, 1004–1017 (2020). 31981247 10.1002/pro.3831 PMC7096713

[21] J. Govaert , Dual beneficial effect of interloop disulfide bond for single domain antibody fragments. J. Biol. Chem. 287, 1970–1979 (2012). 22128183 10.1074/jbc.M111.242818 PMC3283254

[22] X. Tian , Potent binding of 2019 novel coronavirus spike protein by a SARS coronavirus-specific human monoclonal antibody. Emerg. Microbes Infect. 9, 382–385 (2020). 32065055 10.1080/22221751.2020.1729069 PMC7048180

[23] D. F. Robbiani , Convergent antibody responses to SARS-CoV-2 in convalescent individuals. Nature 584, 437–442 (2020). 32555388 10.1038/s41586-020-2456-9 PMC7442695

[24] J. Huo , Neutralizing nanobodies bind SARS-CoV-2 spike RBD and block interaction with ACE2. Nat. Struct. Mol. Biol. 27, 846–854 (2020). 32661423 10.1038/s41594-020-0469-6

[25] Y. Sun, M. Ho, Emerging antibody-based therapeutics against SARS-CoV-2 during the global pandemic. Antib. Ther. 3, 246–256 (2020). 33912795 10.1093/abt/tbaa025 PMC7717131

[26] D. Wrapp ; VIB-CMB COVID-19 Response Team, Structural basis for potent neutralization of betacoronaviruses by single-domain camelid antibodies. Cell 181, 1004–1015 (2020). 32375025 10.1016/j.cell.2020.04.031 PMC7199733

[27] P. Radvak , SARS-CoV-2 B.1.1.7 (alpha) and B.1.351 (beta) variants induce pathogenic patterns in K18-hACE2 transgenic mice distinct from early strains. *Nat. Commun.* **12**, 6559 (2021). 10.1038/s41467-021-26803-w PMC8589842 34772941

[28] N. S. Laursen , Universal protection against influenza infection by a multidomain antibody to influenza hemagglutinin. Science 362, 598–602 (2018). 30385580 10.1126/science.aaq0620 PMC6241527

[29] G. Van Heeke , Nanobodies® as inhaled biotherapeutics for lung diseases. Pharmacol. Ther. 169, 47–56 (2017). 27373507 10.1016/j.pharmthera.2016.06.012

[30] M. Feng , Construction and next-generation sequencing analysis of a large phage-displayed V_NAR_ single-domain antibody library from six naïve nurse sharks. Antib. Ther. 2, 1–11 (2019). 10.1093/abt/tby011 PMC6312525 30627698

[31] M. Ho, R. J. Kreitman, M. Onda, I. Pastan, In vitro antibody evolution targeting germline hot spots to increase activity of an anti-CD22 immunotoxin. J. Biol. Chem. 280, 607–617 (2005). 15491997 10.1074/jbc.M409783200

[32] H. Kim, M. Ho, Isolation of antibodies to heparan sulfate on glypicans by phage display. Curr. Protoc. Protein Sci. 94, e66 (2018). 30091851 10.1002/cpps.66 PMC6205898

[33] Z. Tang , A human single-domain antibody elicits potent antitumor activity by targeting an epitope in mesothelin close to the cancer cell surface. Mol. Cancer Ther. 12, 416–426 (2013). 23371858 10.1158/1535-7163.MCT-12-0731 PMC3624043

[34] M. Letko, A. Marzi, V. Munster, Functional assessment of cell entry and receptor usage for SARS-CoV-2 and other lineage B betacoronaviruses. Nat. Microbiol. 5, 562–569 (2020). 32094589 10.1038/s41564-020-0688-y PMC7095430

[35] C. Z. Chen , Identifying SARS-CoV-2 entry inhibitors through drug repurposing screens of SARS-S and MERS-S pseudotyped particles. ACS Pharmacol. Transl. Sci. 3, 1165–1175 (2020). 33330839 10.1021/acsptsci.0c00112 PMC7586456

[36] T. J. Esparza, N. P. Martin, G. P. Anderson, E. R. Goldman, D. L. Brody, High affinity nanobodies block SARS-CoV-2 spike receptor binding domain interaction with human angiotensin converting enzyme. Sci. Rep. 10, 22370 (2020). 33353972 10.1038/s41598-020-79036-0 PMC7755911

[37] C. Z. Chen , Drug repurposing screen for compounds inhibiting the cytopathic effect of SARS-CoV-2. Front. Pharmacol. 11, 592737 (2021). 33708112 10.3389/fphar.2020.592737 PMC7942396

[38] D. Esposito , Optimizing high-yield production of SARS-CoV-2 soluble spike trimers for serology assays. Protein Expr. Purif. 174, 105686 (2020). 32504802 10.1016/j.pep.2020.105686 PMC7271859

[39] E. Krieger, G. Vriend, YASARA View - molecular graphics for all devices - from smartphones to workstations. Bioinformatics 30, 2981–2982 (2014). 24996895 10.1093/bioinformatics/btu426 PMC4184264

[40] J. Yang, Y. Zhang, I-TASSER server: New development for protein structure and function predictions. Nucleic Acids Res. 43, W174–W181 (2015). 25883148 10.1093/nar/gkv342 PMC4489253

[41] R. Das, D. Baker, Macromolecular modeling with Rosetta. Annu. Rev. Biochem. 77, 363–382 (2008). 18410248 10.1146/annurev.biochem.77.062906.171838

[42] R. E. Cachau , Solution structure of taxol determined using a novel feedback-scaling procedure for Noe-restrained molecular dynamics. Int. J. Supercomput. Appl. High Perform. Comput. 8, 24–34 (1994).

[43] S. Yokoyama , A novel pathway of LPS uptake through syndecan-1 leading to pyroptotic cell death. eLife 7, e37854 (2018). 30526845 10.7554/eLife.37854 PMC6286126

[44] M. J. Abraham , GROMACS: High performance molecular simulations through multi-level parallelism from laptops to supercomputers. SoftwareX 1–2, 19–25 (2015).

[45] R. Henderson , Controlling the SARS-CoV-2 spike glycoprotein conformation. Nat. Struct. Mol. Biol. 27, 925–933 (2020). 32699321 10.1038/s41594-020-0479-4 PMC8581954

[46] B. G. Pierce , ZDOCK server: Interactive docking prediction of protein-protein complexes and symmetric multimers. Bioinformatics 30, 1771–1773 (2014). 24532726 10.1093/bioinformatics/btu097 PMC4058926

[47] M. Igaev, C. Kutzner, L. V. Bock, A. C. Vaiana, H. Grubmüller, Automated cryo-EM structure refinement using correlation-driven molecular dynamics. eLife 8, e43542 (2019). 30829573 10.7554/eLife.43542 PMC6424565

[48] E. F. Pettersen , UCSF Chimera—A visualization system for exploratory research and analysis. J. Comput. Chem. 25, 1605–1612 (2004). 15264254 10.1002/jcc.20084

[49] Y. Yuan , Cryo-EM structures of MERS-CoV and SARS-CoV spike glycoproteins reveal the dynamic receptor binding domains. Nat. Commun. 8, 15092 (2017). 28393837 10.1038/ncomms15092 PMC5394239

[50] D. Liebschner , Macromolecular structure determination using X-rays, neutrons and electrons: Recent developments in Phenix. Acta Crystallogr. D Struct. Biol. 75, 861–877 (2019). 31588918 10.1107/S2059798319011471 PMC6778852

