# Supplementary material for: Dromedary camel nanobodies broadly neutralize SARS-CoV-2 variants
Source: Proc Natl Acad Sci U S A. 2022 Apr 27;119(18):e2201433119. doi: 10.1073/pnas.2201433119 (PMC9170159; doi:10.1073/pnas.2201433119)
Supplement: Supplementary File [file pnas.2201433119.sapp.pdf]

PNAS
www.pnas.org

Supplementary Information for
Dromedary camel nanobodies broadly neutralize SARS-CoV-2 variants

Jessica Hong[1#], Hyung Joon Kwon[2#], Raul Cachau[3#], Catherine Z. Chen[4], Kevin John Butay[5], Zhijian Duan[6], Dan Li[1], Hua Ren[1], Tianyuzhou Liang[1], Jianghai Zhu[3], Venkata P. Dandey[5], Negin Martin[7], Dominic Esposito[8], Uriel Ortega-Rodriguez[2], Miao Xu[4], Mario J. Borgnia[5]*, Hang Xie[2]*, Mitchell Ho[1,6]*

Correspondence to: homi@mail.nih.gov (Lead correspondence)

mario.borgnia2@nih.gov

Hang.Xie@fda.hhs.gov

This PDF file includes:

Figs. S1 to S15
Tables S1 to S5

```
7A3       QVQLVESGGGSVQPGGSLRLSCVVSGYTSSSRYMGWFRQVPGKGLEWVSGIKRDGTNTYYADSVKGR
8A4       EVQLVESGGGLVQPGESLRLSCEASGFTFSSVYMSWVRQAPGKGLEWISTIHPAGGSTYYADSMKDR
1B5       DVQLVESGGGSVQAGGSLRLSCTGSRYTYSTYCMGWFRQAPGKEEEAVAIINSGGGEPYYGDSVKGRF
8A2       AVQLVDSGGGSVQAGGSLRLSCAASGYTYSICTMGWYRQAPGEGLEWVSGINADGSNTHYTDSVKGR
2F7       QVKLEESGGGSVQSGGSLRLSCTVSRDTNTNINRCMGWFRQAPGKGLETVATINRDGTNTYYTDAVKGR
1H6       AVQLVDSGGGSVQAGGSLNLSCVASGTTLRNGCMAWFRQVPGKEREVVAIIIRATSYTDYADSVKGR


7A3       FTISQDNAKNTVYLQMNSLKPEDTAMYYCAAGSWYNQWGYSMDYWGKGTQVTVSS        122
8A4       FTISRDNAKNTLYLQMNSLKSEDTALYYCIIEALSGYRGPGTQVTVSS               115
1B5       FTISQDRAKNTVYLQMDGLQPDDTAIYYCVAADSHNSRCYLGRSYVNYWGQGTQVTVSS    126
8A2       FTISRDNAKKTLYLQMNSLKPEDTAIYYCAAHGTYDKYAPCGGFAGTYTYWGQGTQVTVSS  128
2F7       FTISQDNVKNTVYLQMNNLTPEDTGTYICNAMGRGSGSRCDNWDPNYWGQGTQVTVSS     127
1H6       FTISQDNAKNTVYLQMKSLTPEDTATYYCAATLYRVNCAKREFDKWGQGTQVTVSS       123
```

IMGT, *Kabat*, <u>Paratome</u>

Fig. S1. Sequence alignment of the top 6 V_HH with CDR regions bolded for IMGT, italicized for Kabat, and underlined for Paratome. The cysteines are highlighted in red. All the V_HH sequences are also listed in provisional patent application no. PCT/US2021/056548.

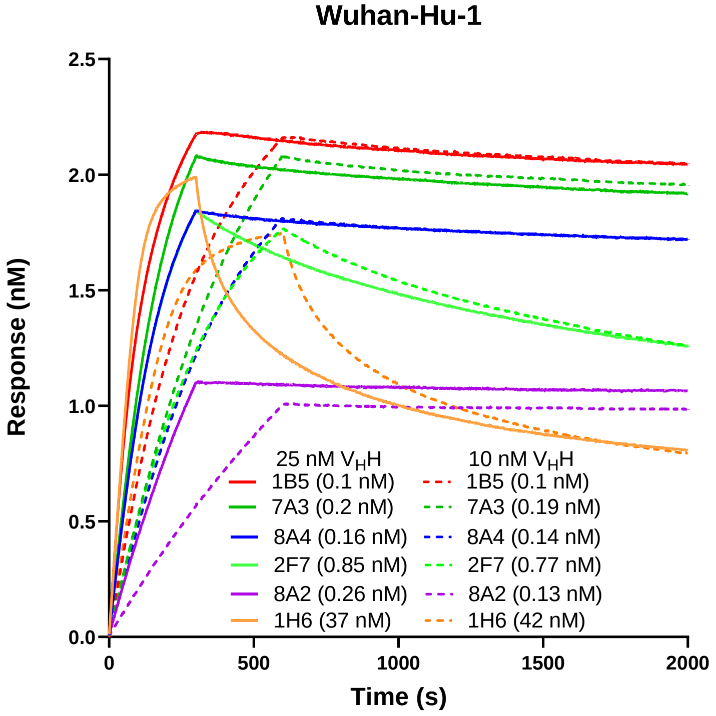

V_HH	Conc. (nM)	KD (M)	KD Error	Kon (1/Ms)	Kon Error	Kdis (1/s)	Kdis Error
1B5	25	1.00E-10	<1E-12	3.40E+05	5.40E+02	3.70E-05	<1e-7
7A3	25	2.00E-10	1.13E-12	2.00E+05	5.30E+02	4.30E-05	1.50E-07
8A4	25	1.80E-10	1.00E-12	2.32E+05	5.20E+02	3.70E-05	4.50E-07
2F7	25	8.50E-10	6.70E-12	2.60E+05	1.80E+03	2.20E-04	7.20E-07
8A2	25	2.60E-10	1.80E-12	7.40E+04	2.40E+02	1.90E-05	3.60E-07
1H7	25	3.70E-08	2.00E-12	8.30E+05	2.50E+03	5.40E-03	5.20E-07
1B5	10	1.10E-10	<1e-12	3.20E+05	3.00E+02	3.40E-05	<1e-7
7A3	10	1.90E-10	1.10E-12	2.00E+05	4.70E+02	3.80E-05	6.00E-07
8A4	10	1.40E-10	<1e-12	2.40E+05	4.50E+02	3.40E-05	2.70E-07
2F7	10	7.70E-10	5.00E-12	3.00E+05	1.60E+03	2.20E-04	3.20E-07
8A2	10	1.30E-10	1.00E-12	8.70E+04	1.70E+02	1.20E-05	1.50E-07
1H7	10	4.20E-08	1.30E-12	8.30E+05	1.60E+03	5.40E-03	8.70E-07

Fig. S2. The binding avidity of V_HH-hFcs against the RBD of Wuhan-Hu-1. The K_D values were measured on Octet using two concentrations (10 and 25 nM) of the V_HH-hFc protein. Conc, concentration.

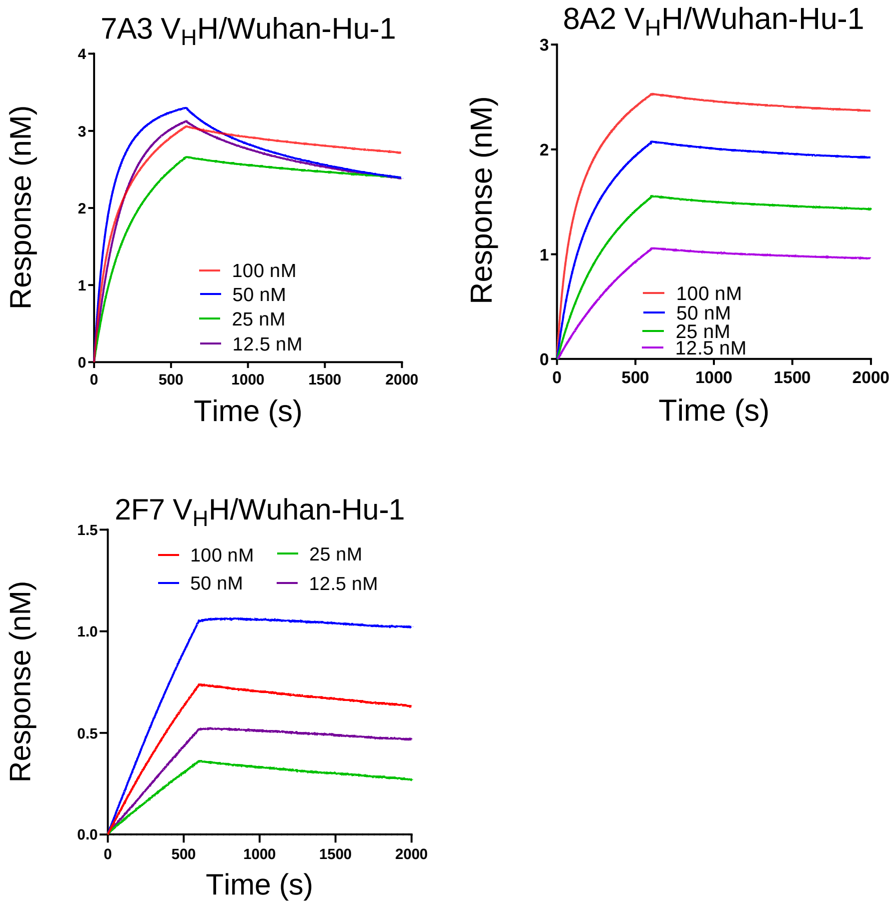

V_HH	Conc. (nM)	KD (M)	KD Error	Kon (1/Ms)	Kon Error	Kdis (1/s)	Kdis Error
7A3	100	7.00E-10	1.03E-11	6.20E+04	2.20E+02	4.30E-05	6.20E-07
7A3	50	4.30E-10	3.60E-12	8.20E+04	1.55E+02	3.50E-05	2.90E-07
7A3	25	5.00E-10	1.30E-12	9.50E+04	1.20E+02	6.70E-05	1.40E-07
7A3	12.5	1.70E-10	<1e-12	1.00E+05	1.64E+03	1.70E-05	<1e-7
8A2	100	2.41E-10	1.11E-11	6.32E+04	2.53E+02	1.52E-05	6.95E-07
8A2	50	7.64E-10	4.41E-12	8.96E+04	2.00E+02	6.85E-05	3.65E-07
8A2	25	8.05E-10	2.31E-12	1.10E+05	1.83E+02	8.85E-05	2.07E-07
8A2	12.5	1.30E-09	5.81E-12	9.45E+04	3.77E+02	1.23E-04	2.49E-07
2F7	100	1.70E-09	1.30E-11	9.70E+04	4.90E+02	1.70E-04	9.50E-07
2F7	50	1.20E-09	8.10E-12	1.10E+05	4.60E+02	1.40E-04	7.40E-07
2F7	25	7.50E-10	4.40E-12	1.20E+05	3.80E+02	8.90E-05	4.20E-07
2F7	12.5	3.00E-10	1.00E-12	1.00E+05	1.50E+02	3.00E-05	<1e-7

Fig. S3. The binding affinity of camel V_HHs against the RBD of Wuhan-Hu-1 on Octet. The 7A3, 8A2, and 2F7 V_HHs were expressed in *E. coli* and purified on a nickel column. The binding affinity of the camel V_HHs on the spike protein of Wuhan-Hu-1 was measured on Octet using the RBD protein. Conc, concentration.

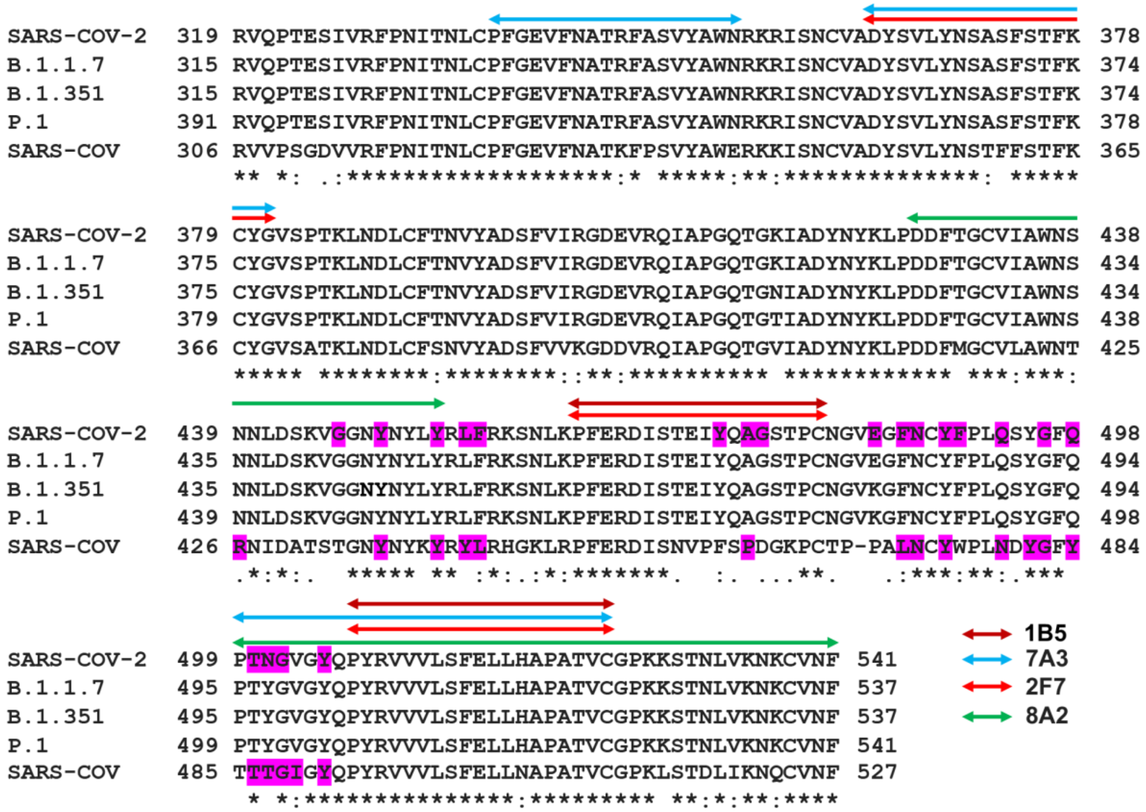

Fig. S4. Epitope mapping of anti-SARS-CoV-2 RBD V_HHs. Sequence alignment of RBD region of SARS-CoV-2, SARS-CoV, and three identified variant strains of SARS-CoV-2 (B.1.1.7 EPI_ISL_601443, B.1.351 EPI_ISL_700428, and P.1 EPI_ISL_792680). The conserved residues are marked with asterisks (*), the residues with similar properties between variants are marked with the colon symbol (:), and the residues with marginally similar properties are marked with the period symbol (.). The main residues of both SARS-CoV-2 and SARS-CoV RBD region identified previously that interact with ACE2 are shaded in magenta. Arrows of different lengths and colors represent possible positions of V_HH contact on the RBD.

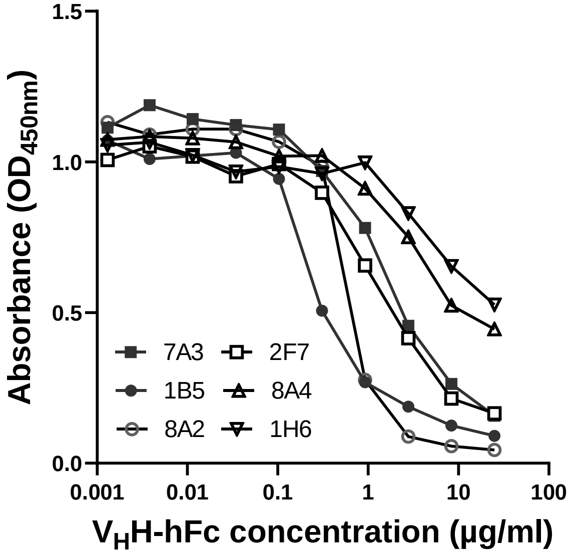

Fig. S5. Inhibition effect of V_HH nanobodies against the interaction of the RBD and the human ACE2 protein by ELISA.

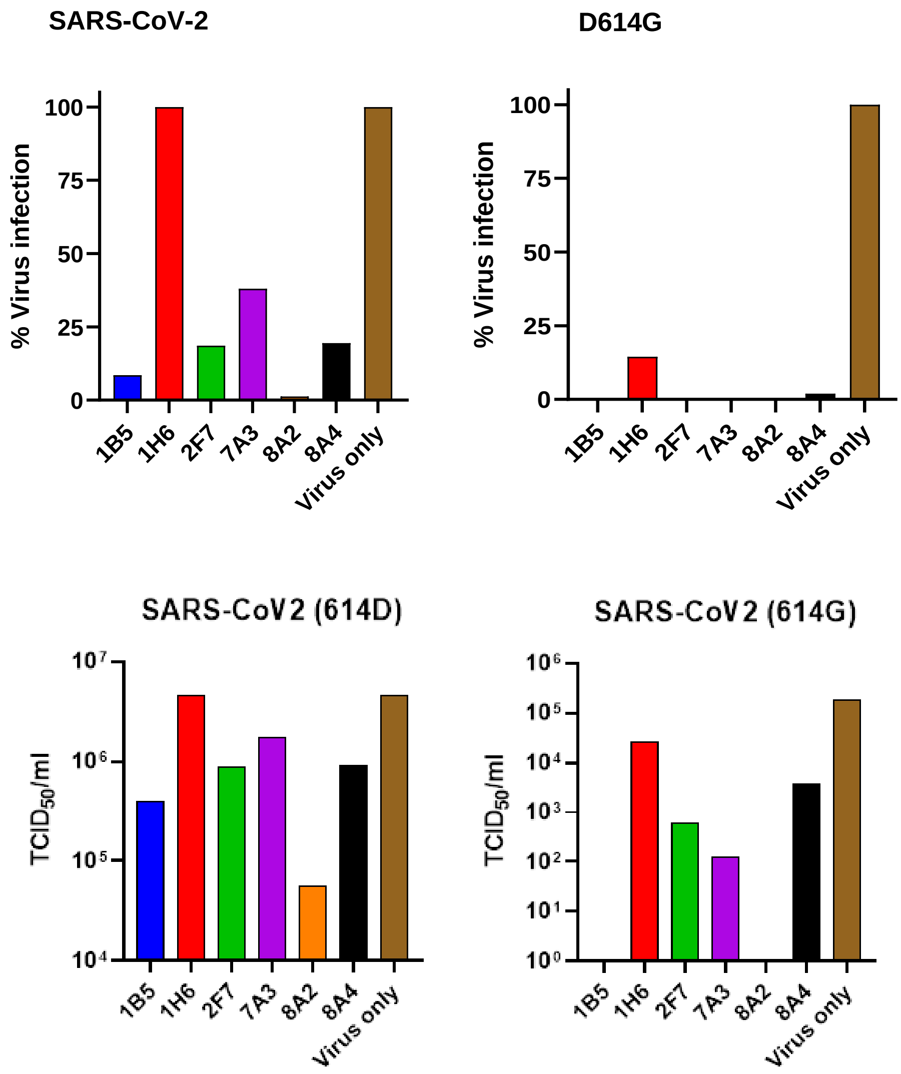

Fig. S6. Prescreening V_HH-hFc fusion proteins at 10 µg/ml against live SARS-CoV-2 bearing 614D or 614G. V_HH-hFc fusion proteins at the concentration of 10 µg/ml were incubated with 10^2 $TCID_{50}$ of live SARS-CoV-2 or the 614G variant at room temperature for 1 h. Then, the mixtures were added to Vero E6 cells pre-seeded in 96-well flat-bottom tissue culture plates and incubated at 37°C, 5% CO_2 for 72 h. Residual virus in the supernatants was titrated by $TCID_{50}$ assay.

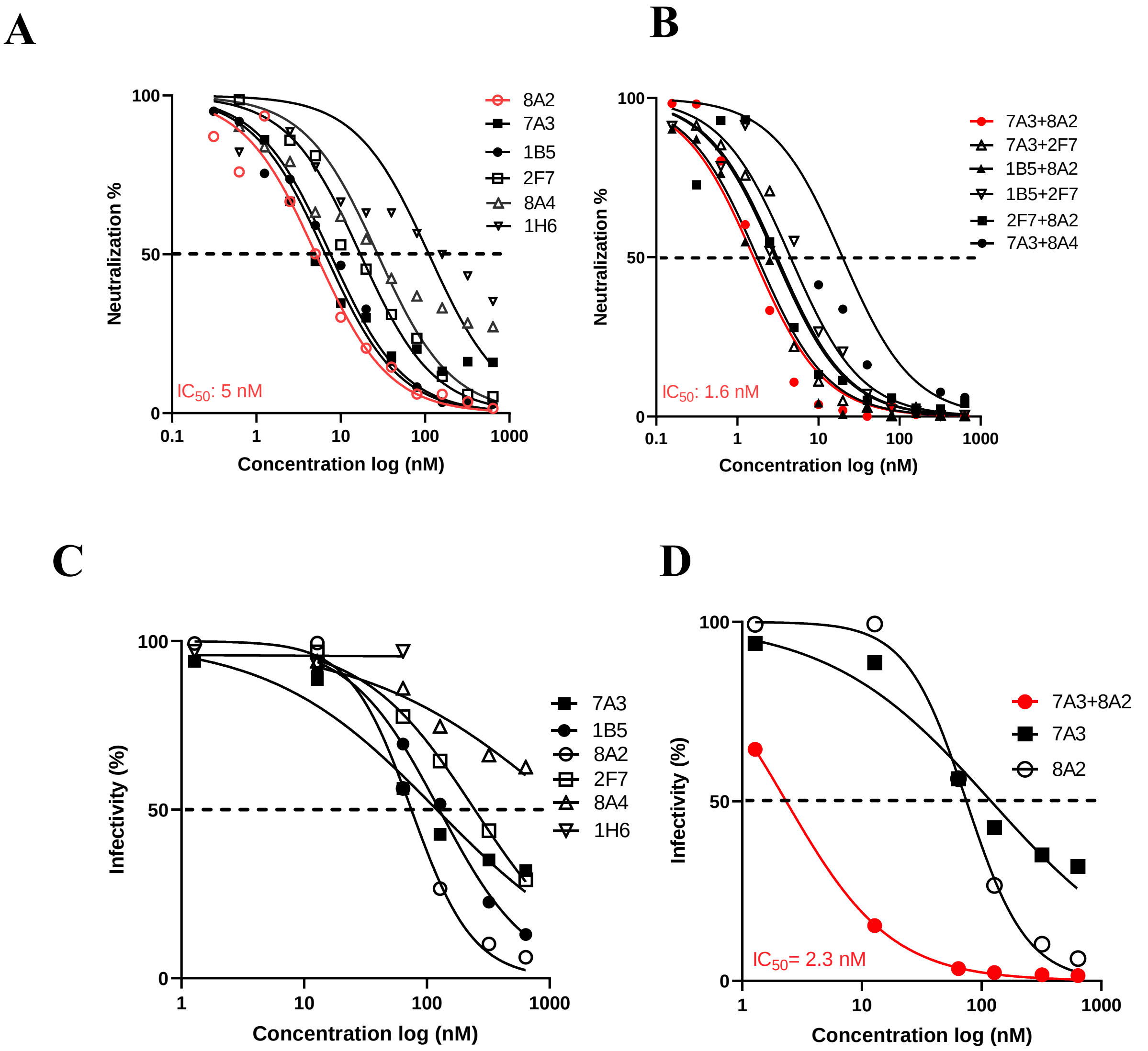

Fig. S7. Neutralization assay on SARS-CoV-2 (Wuhan-Hu-1). **A, B**) Lentivirus-based pseudovirus assays. **C, D**) Fluorescence reporter assays.

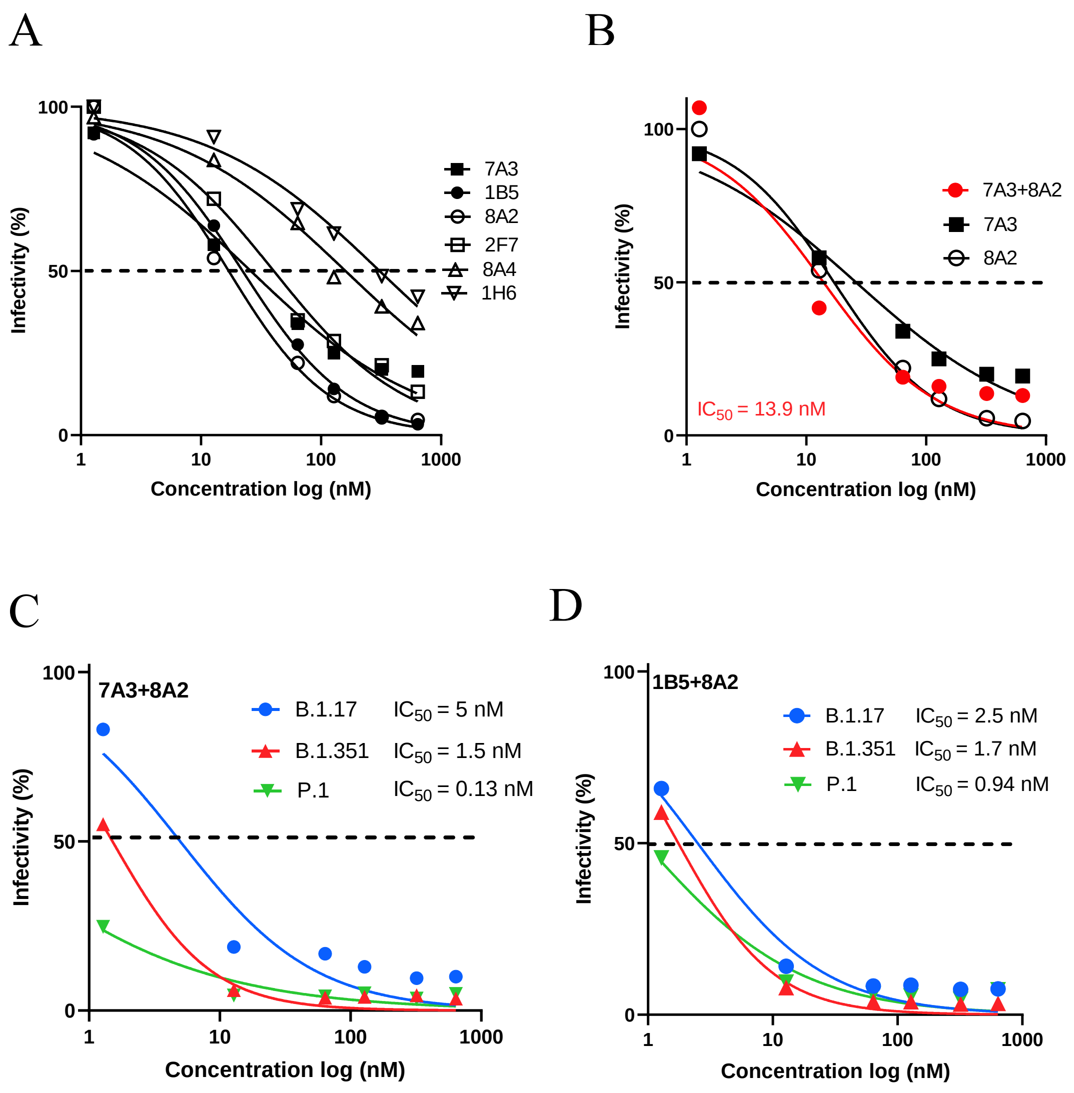

Fig. S8. Neutralization of the variants of SARS-CoV-2 pseudoviral infection. **A, B)** Fluorescence reporter assays using D614G variant. **C, D**) Fluorescence report assays using B.1.1.7, B.1.351, and P.1 variants.

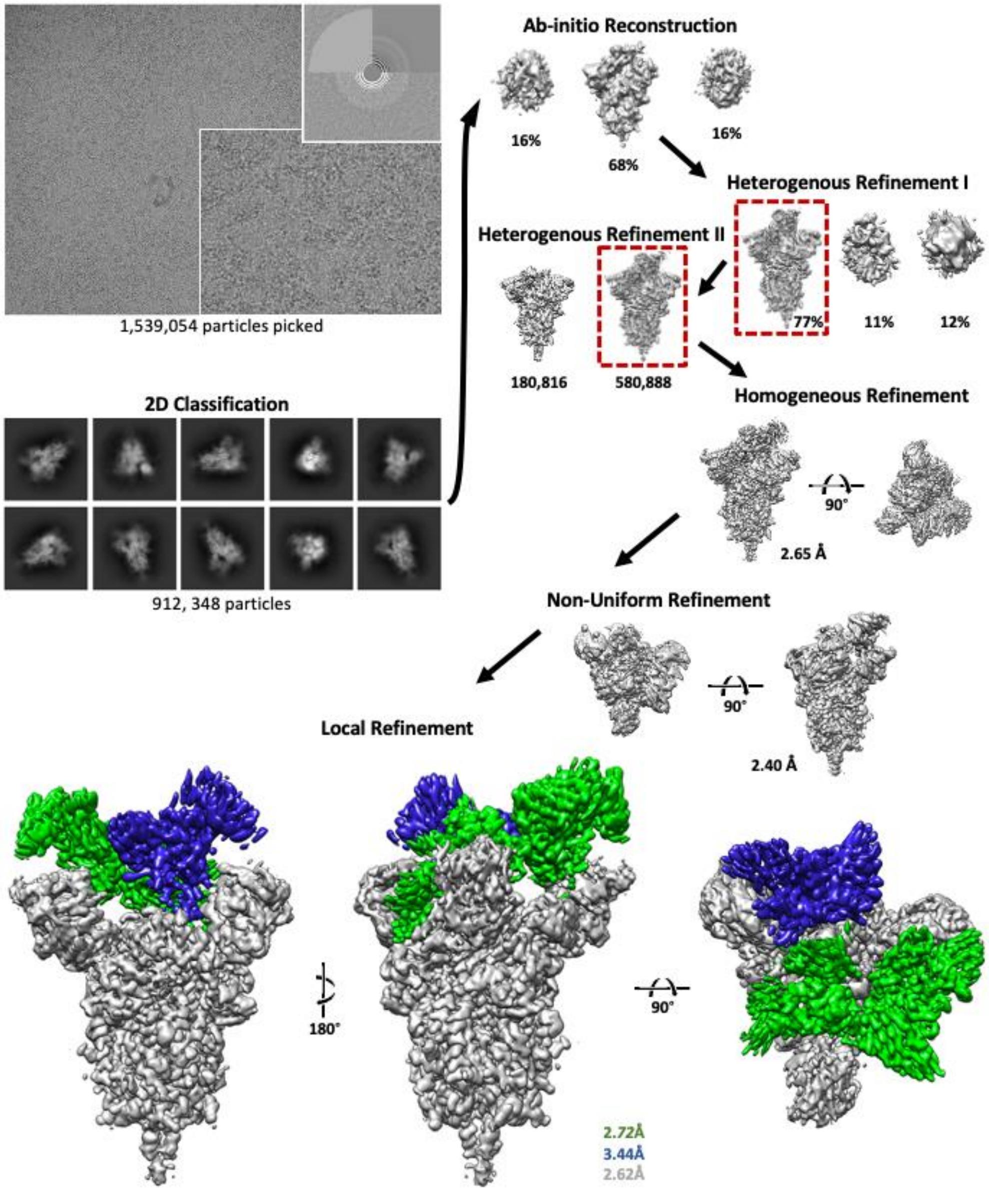

Fig. S9. Cryo-EM image processing workflow for S-protein ectodomain in complex with 7A3 and 8A2. A total of 1,539,054 particles selected from 4,644 micrographs were subject to 2D classification. Class selection yielded 912,348, which were used to generate three initial maps by Ab-initio reconstruction, followed by two cycles of heterogenous refinement and a third non-uniform refinement step, and a final refinement focused on the RBDs.

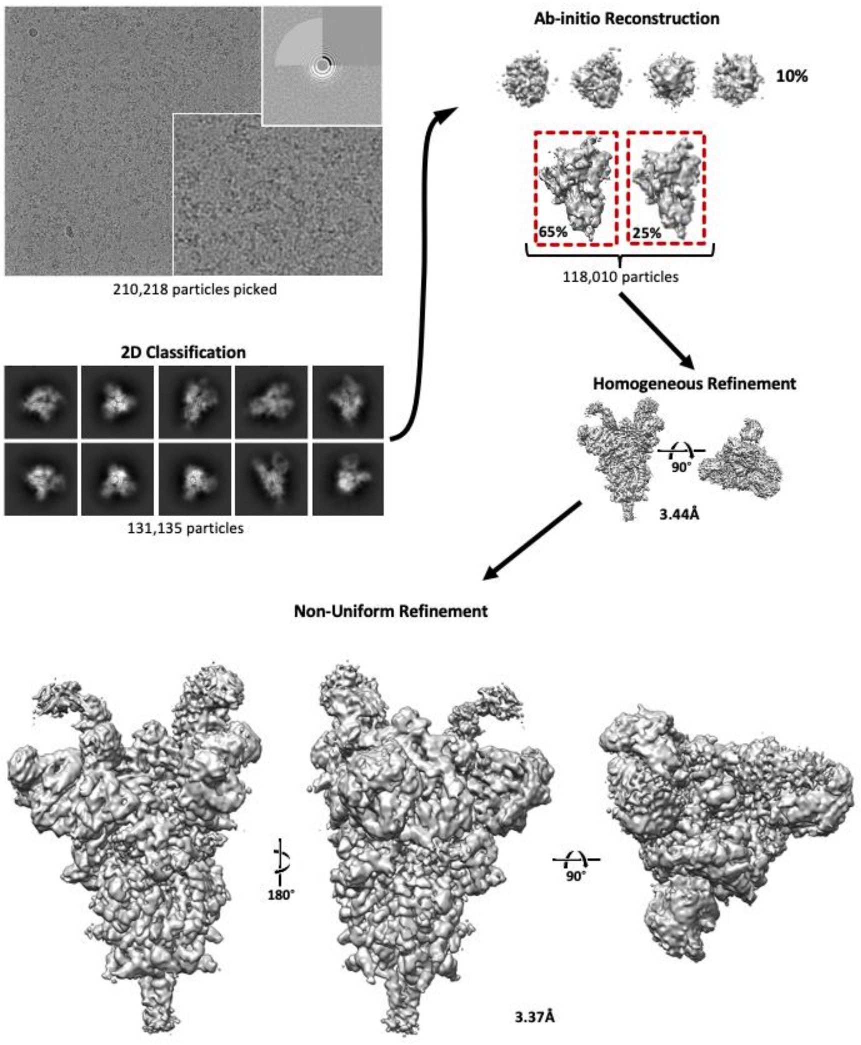

Fig. S10. Cryo-EM image processing workflow for S-protein ectodomain in complex with 8A2. A total of 210,218 particles selected from 2149 micrographs were subject to 2D classification. Class selection yielded 131,135 which were used to generate three initial maps by Ab-initio reconstruction, followed by heterogeneous refinement and non-uniform refinement.

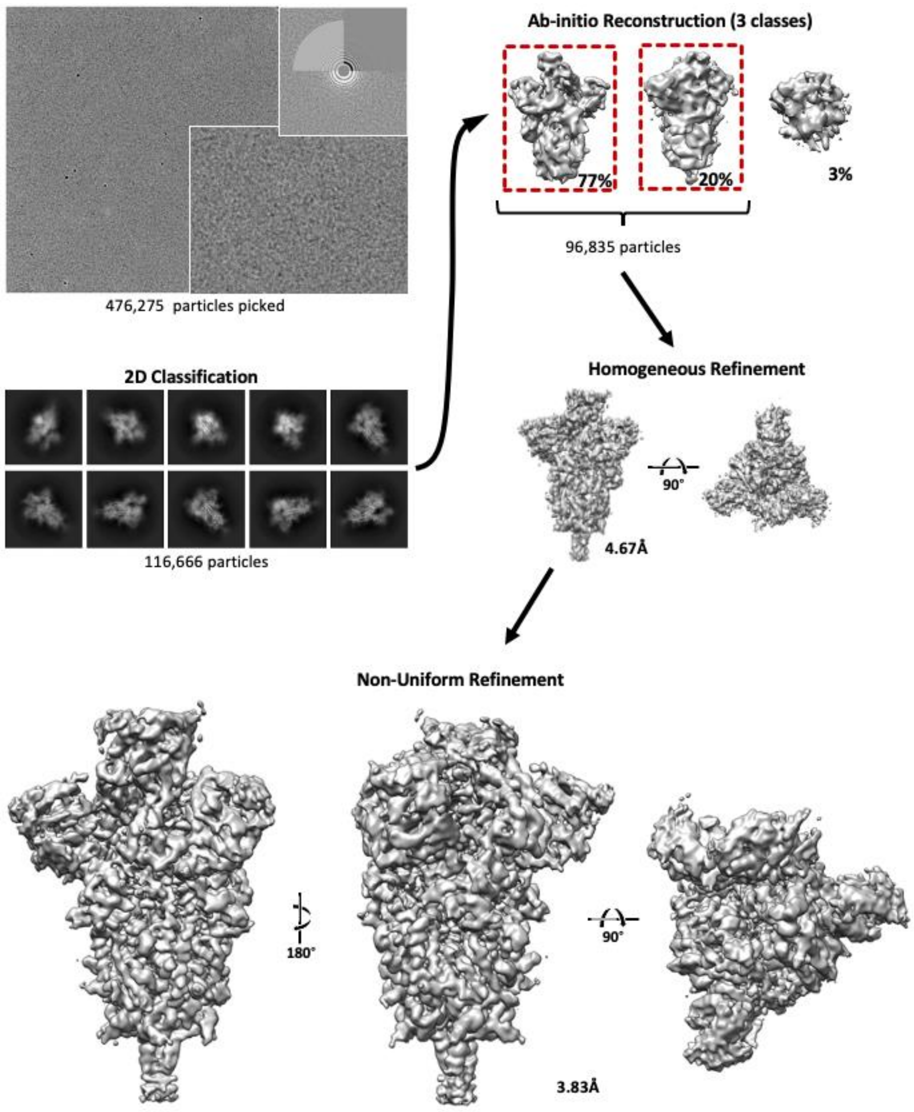

Fig. S11. Cryo-EM image processing workflow for S-protein ectodomain in complex with 7A3. A total of 476,275 particles selected from 3,198 micrographs were subject to 2D classification. Class selection yielded 116,666, which were used to generate three initial maps by Ab-initio reconstruction, followed by heterogeneous refinement and non-uniform refinement.

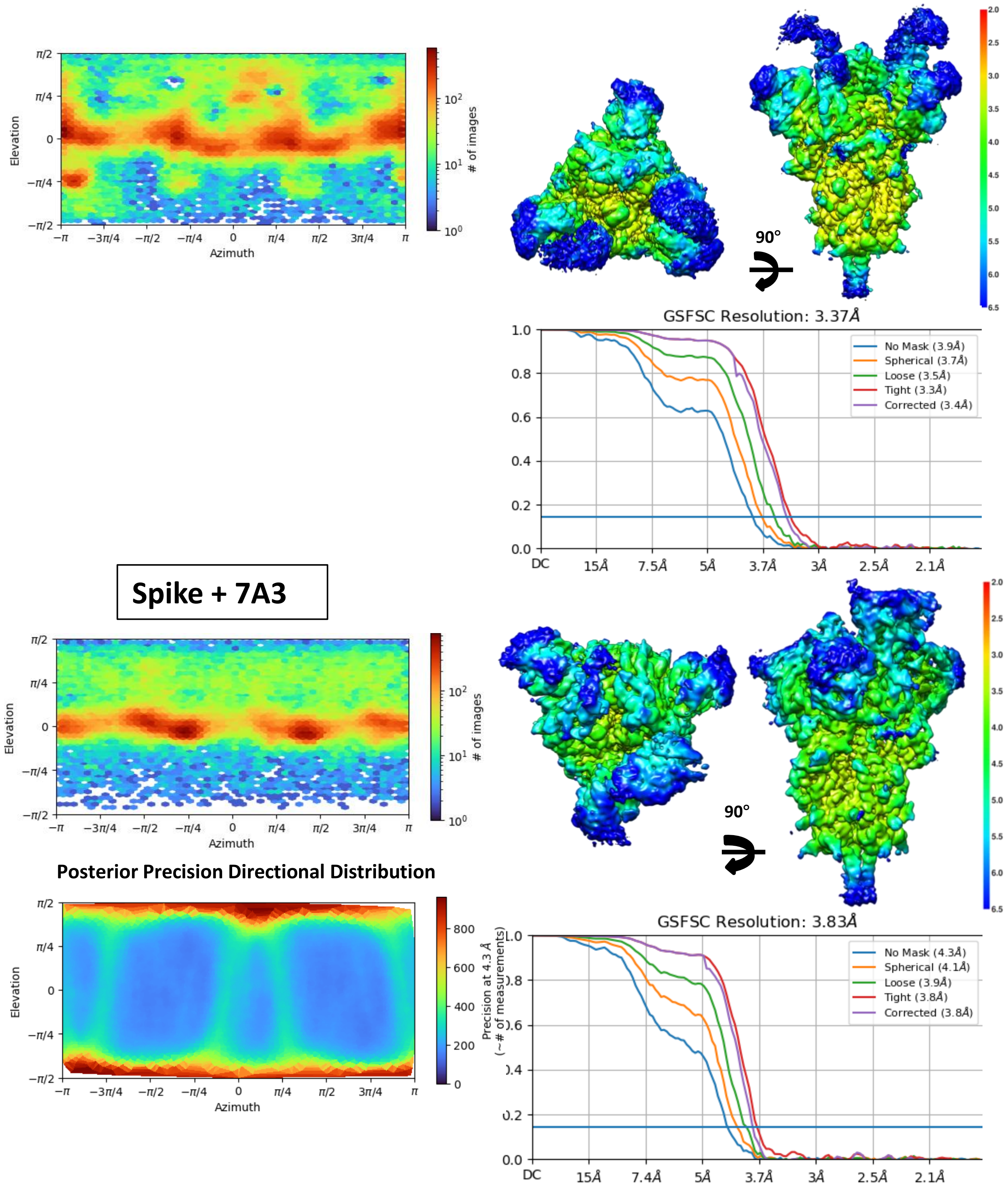

Fig. S12. Image Processing Statistics of the Cryo-EM maps for the interaction of SARS-CoV-2 spike protein with 8A2 or 7A3 V_HH.

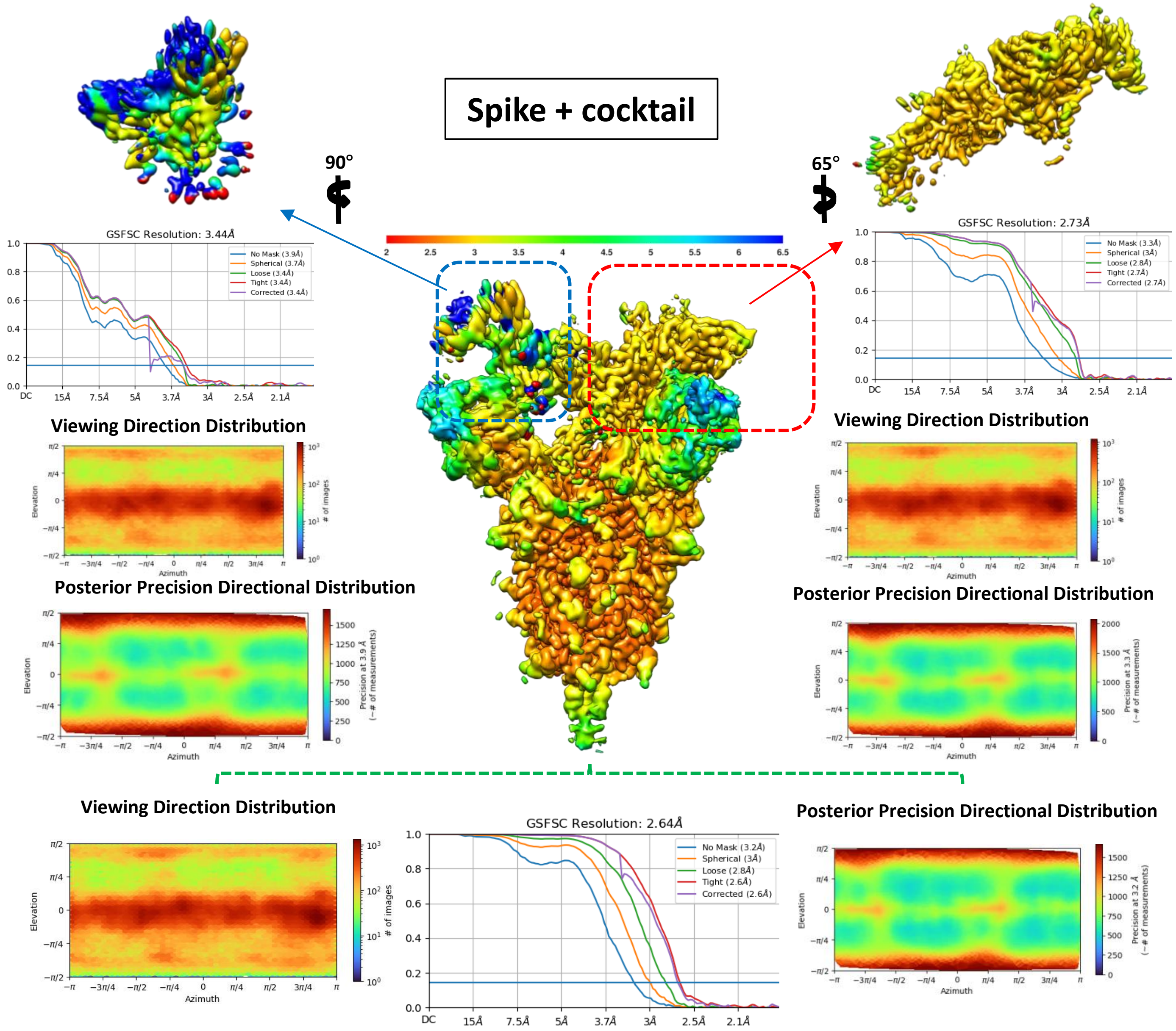

Fig. S13. Image Processing Statistics of the Cryo-EM map for the interaction of SARS-CoV-2 spike protein with 8A2 and 7A3 $V_H H$ nanobody cocktail.

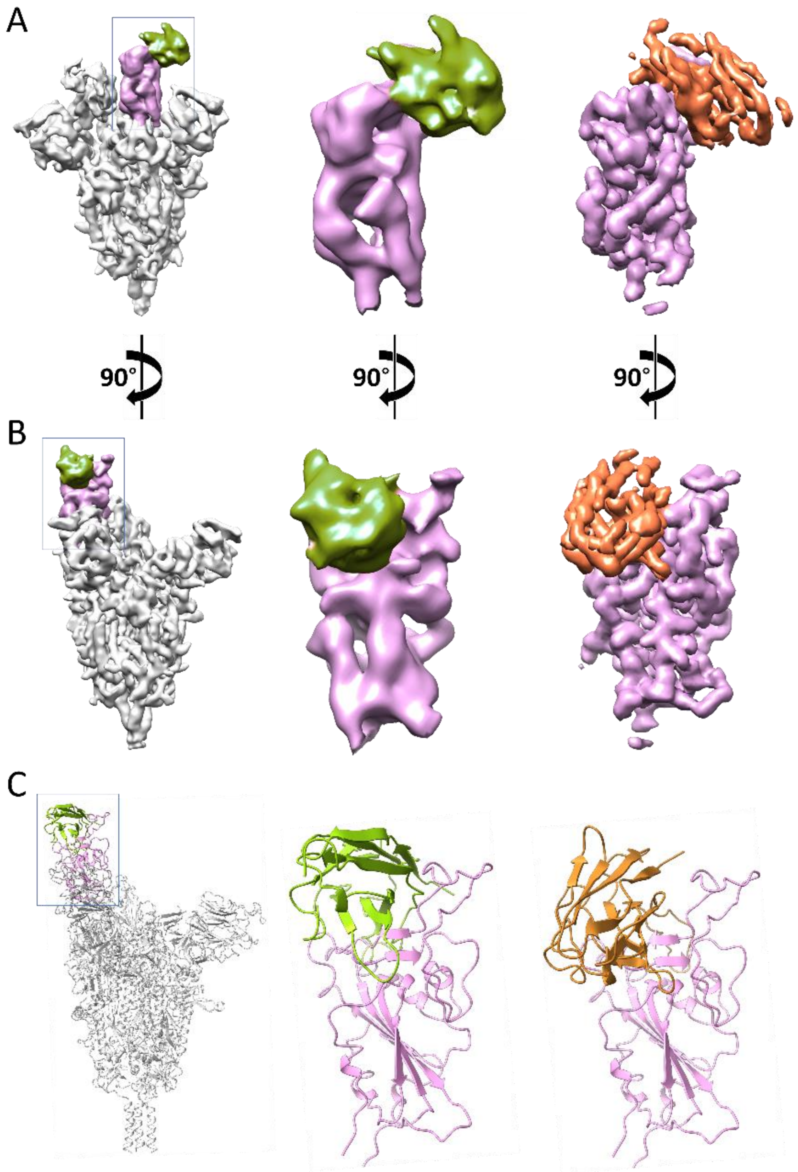

Fig. S14. The 2F7 V_HH/spike map and its comparison to the 8A2 V_HH/spike complex structure. **A**) From left to right, the first panel shows the 2F7 V_HH/spike complex map, RBD is colored purple and 2F7 in green. The second panel shows an enlarged section indicated in the first panel with a bounding box in light blue; the third panel shows the RBD-8A2 complex map in a similar orientation as the middle panel. **B**) Same as A with the structures rotated 90° clockwise around the vertical axis. **C**) Structural models of 2F7 and 8A2 complexes in the same orientation as in B. The 2F7 map resolution is insufficient to resolve the features of the nanobody but unambiguously identifies the general epitope area, confirming 2F7 shares the same binding loci as 8A2. A refined structure of the 2F7 complex cannot be generated due to the low resolution of the map. 2F7 pose in panel C was generated by manually correcting the overall fitting of a 2F7 model to the map using the program Chimera version 1.14 using a homologous model of 2F7 generated from the coordinates of 8A2.

[Q]CVNLTTRTQLPPAYTNSFTRGVYYPDKVFRSSVLHSTQDLFLPFFSNVTWFHA
IHVSGTNGTKRFDNPVLPFNDGVYFASTEKSNIIRGWIFGTTLDSKTQSLLIVNNA
TNVVIKVCEFQFCNDPFLGVYYHKNNKSWMESEFRVYSSANNCTFEYVSQPFLM
DLEGKQGNFKNLREFVFKNIDGYFKIYSKHTPINLVRDLPQGFSALEPLVDLPIGI
NITRFQTLLALHRSYLTPGDSSSGWTAGAAAYYVGYLQPRTFLLKYNENGTITDA
VDCALDPLSETKCTLKSFTVEKGIYQTSNFRVQPTESIVRFPNITNLCPFGEVFNAT
RFASVYAWNRKRISNCVADYSVLYNSASFSTFKCYGVSPTKLNDLCFTNVYADS
FVIRGDEVRQIAPGQTGKIADYNYKLPDDFTGCVIAWNSNNLDSKVGGNYNYLY
RLFRKSNLKPFERDISTEIYQAGSTPCNGVEGFNCYFPLQSYGFQPTNGVGYQPY
RVVVLSFELLHAPATVCGPKKSTNLVKNKCVNFNFNGLTGTGVLTESNKKFLPF
QQFGRDIADTTDAVRDPQTLEILDITPCSFGGVSVITPGTNTSNQVAVLYQDVNCT
EVPVAIHADQLTPTWRVYSTGSNVFQTRAGCLIGAEHVNNSYECDIPIGAGICAS
YQTQTNSPGSASSVASQSIIAYTMSLGAENSVAYSNNSIAIPTNFTISVTTEILPVS
MTKTSVDCTMYICGDSTECSNLLLQYGSFCTQLNRALTGIAVEQDKNTQEVFAQ
VKQIYKTPPIKDFGGFNFSQILPDPSKPSKRS**P**IEDLLFNKVTLADAGFIKQYGDCL
GDIAARDLICAQKFNGLTVLPPLLTDEMIAQYTSALLAGTITSGWTFGAG**P**ALQIP
F**P**MQMAYRFNGIGVTQNVLYENQKLIANQFNSAIGKIQDSLSST**P**SALGKLQDVV
NQNAQALNTLVKQLSSNFGAISSVLNDILSRLD**PP**EAEVQIDRLITGRLQSLQTYV
TQQLIRAAEIRASANLAATKMSECVLGQSKRVDFCGKGYHLMSFPQSAPHGVVF
LHVTYVPAQEKNFTTAPAICHDGKAHFPREGVFVSNGTHWFVTQRNFYEPQIITT
DNTFVSGNCDVVIGIVNNTVYDPLQPELDSFKEELDKYFKNHTSPDVDLGDISGI
NASVVNIQKEIDRLNEVAKNLNESLIDLQELGKYEQGSGYIPEAPRDGQAYVRKD
GEWVLLSTFLGRSLEVLFQGPGHHHHHHHHSAWSHPQFEKGGGSGGGGSGGSA
WSHPQFEK

Fig. S15. The protein sequence of stabilized SARS-CoV-2 S protein used for the cryo-EM V_HH complex structures in the present study. N-terminus is a cyclized glutamine (pyroglutamic acid). Six proline substitutions are bold in red.

V_HH-Fc	Wuhan-Hu-1	D614G	N501Y	B.1.1.7	B.1.351	P.1	B.1.617.2
	KD (nM)						
8A2	0.26	0.2	0.11	0.001	0.001	0.5	NB
1B5	0.1	0.2	0.12	0.03	0.26	0.5	0.14
7A3	0.2	0.23	0.25	0.2	0.1	0.8	0.42
2F7	0.85	1	0.85	3.2	NB	NB	NB
8A4	0.16	0.6	0.5	5.6	NB	NB	NB
1H6	37	23	47	17	NB	NB	NB

	V_HH-Fc	KD Error	Kon (1/Ms)	Kon Error	Kdis (1/s)	Kdis Error
Wuhan-Hu-1	7A3	1.13E-12	2.23E+05	5.31E+02	4.34E-05	2.30E-07
	1B5	1.64E-12	3.02E+05	7.79E+02	6.70E-05	4.65E-07
	2F7	6.67E-12	2.59E+05	1.78E+03	2.19E-04	8.42E-07
	8A2	1.77E-12	7.38E+04	2.37E+02	1.93E-05	1.15E-07
	8A4	1.02E-12	2.32E+05	5.19E+02	3.74E-05	2.21E-07
	1H6	2.09E-11	8.26E+05	2.50E+04	5.36E-04	5.90E-06
B.1.1.7	7A3	1.56E-12	3.18E+05	8.00E+02	7.94E-05	4.54E-07
	1B5	1.13E-12	3.49E+05	7.68E+02	4.12E-05	3.83E-07
	2F7	8.58E-12	3.72E+05	3.14E+03	3.17E-04	1.74E-06
	8A4	2.69E-12	1.99E+05	5.34E+02	9.80E-05	4.66E-07
	8A2	1.33E-12	9.95E+04	1.19E+02	1.13E-05	1.31E-07
	1H6	1.39E-11	9.35E+05	2.58E+04	4.43E-04	4.43E-06
B.1.351	7A3	6.42E-12	1.64E+05	7.04E+02	1.30E-04	8.94E-07
	1B5	3.04E-12	2.81E+05	7.14E+02	1.33E-04	7.83E-07
	8A2	7.80E-12	7.05E+04	3.84E+02	3.70E-05	5.12E-07
P.1	7A3	6.42E-12	1.64E+05	7.04E+02	1.30E-04	8.94E-07
	1B5	3.04E-12	2.81E+05	7.14E+02	1.33E-04	7.83E-07
	8A2	7.80E-12	7.05E+04	3.84E+02	3.70E-05	5.12E-07
B.1.617.2	7A3	3.09E-12	2.48E+05	8.98E+02	1.07E-04	6.64E-07
	1B5	1.06E-12	2.99E+05	5.19E+02	4.22E-05	3.08E-07

Table S1. Binding of SARS-CoV-2 V_HH-Fc fusions against the RBD of the original Wuhan-Hu-1 and variants on Octet. NB: no binding.

Assay	LP assay	PP entry assay				Fluorescent reporter assay				
V_HH	Wuhan-Hu-1	Wuhan-Hu-1	B.1.1.7	B.1.351	P.1	Wuhan-Hu-1	D614G	B.1.1.7	B.1.351	P.1
	IC_{50} (nM)									
8A2	5	14	2	1	0.5	74.8	17			
1B5	7.6	25		5	5	127	22.2			
7A3	6.6	3	0.6	1	2	246	40.4			
2F7	8.6	15	10			120	26			
8A4	27.4					1388	160			
1H6	111.3						308			
8A2+7A3	1.6	1	0.4	0.3	0.2	2.3	13.9	5	1.5	0.13
8A2+1B5	1.8	5	0.9	0.5	0.3	12.6	14.8	2.5	1.7	0.94
7A3+2F7	2.9	1	0.5	1	5	20.1	17.6	2.5	25.6	3.8
7A3+8A4	19.8					35.7				
8A2+2F7	3.1					40.3	39.8	40	2.6	1.6
1B5+2F7	4.5	4	1	7	2	33.5	28.7	8	14.9	26.1

LP: lentivirus-based pseudovirus
PP: Pseudotyped particle

Table S2. Testing of SARS-CoV-2 V_HH-Fc fusion proteins and combinations against the infection of pseudovirus expressing the original Wuhan-Hu-1 or variant spike protein.

Assay	Live viral microneutralization assay						Cytopathic assay
V_HH	Wuhan-Hu-1	D614G	B.1.1.7	B.1.351	P.1	B.1.617.2	Wuhan-Hu-1
	IC_{50} (nM)						IC_{50} (nM)
8A2	72	23	17	2	0.16	NA	72
1B5	18	12	140	22	1		242
7A3	46	4	25	7	1.4	19	56
2F7	36	13	186	NA	NA		169
8A4							108
1H6							1125
8A2+ 7A3	1	6	2	0.87	0.14	27	20
8A2+ 1B5							47
7A3+ 2F7	1.5	12	5	27	1		16
7A3+ 8A4							35
8A2+ 2F7	15	76	44	3	0.6		97
1B5+ 2F7							40

NA: no activity

Table S3. Testing of SARS-CoV-2 V_HH-hFc fusion proteins and combinations against Wuhan-Hu-1 and emerging variant live viruses. NA, no activity.

Dataset	Hexapro/7A3	Hexapro/8A2	Hexapro/7A3/8A2 I	Hexapro/7A3/8A2 II
Microscope	Arctica	Arctica	Arctica	Krios
Voltage (KeV)	200	200	200	300
Detector	K2	K2	K2	K3
GIF slit (eV)	N/A	N/A	N/A	20
Frames	60	60	60	50
Exposure (s)	9	9	7.8	4.49
Flux (e/A)	6.51	6.18	6.4	3.75
Pixel size	0.932	0.932	0.932	0.528
Mode	Counting	Counting	Counting	Super-Res
Defocus Range	(-1.5, -2.2)	(-1.5, -2.2)	(-1.5, -2.2)	(-1.2, -2.2)

Table S4. Cryo-EM datasets.

Primers	Sequence (5’-3’)
**CamelS1-F**	TACCGTGGCCCAGGCGGCC GAGGTGCAGCTGGTGGAGTCTGG
**CamelS2-F**	TACCGTGGCCCAGGCGGCC CAGGTGCAGCTGGTGGAGTCTGG
**CamelS3-F**	TACCGTGGCCCAGGCGGCC GCGGTGCAGCTGGTGGAGTCTGG
**CamelS4-F**	TACCGTGGCCCAGGCGGCC GATGTGCAGCTGGTGGAGTCTGG
**CamelS5-F**	TACCGTGGCCCAGGCGGCC CAGGTAAAGCTGGAGGAGTCTGG
**CamelS6-F**	TACCGTGGCCCAGGCGGCC GCCGTGCAGCTGGTGGATTCTGG
**CamelVHH-R**	GGTGGCCGGCCTGGCCACT TGAGGAGACGGTGACCTGGGTCC
**Vector-F**	GGCCAGGCCGGCCACCACC
**Vector-R**	GCGAAACCAGCCAGTGCCACTGC
**F-linker**	TACCGTGGCCCAGGCGGCC

Table S5. The primers used to amplify camel V_HH regions, vector backbone, and EASeL overlap extension PCR. Six different forward primers (CamelS1-6F) were used to clone camel V_HH fragments. Vector-F and Vector-R were used for amplification of the pComb3X phagemid vector backbone. F-linker and Vector-R were used in the EASeL overlap extension PCR.
